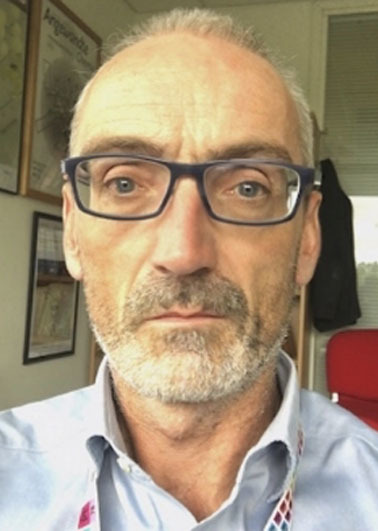# Metathesis by Partner Interchange in σ‐Bond Ligands: Expanding Applications of the σ‐CAM Mechanism

**DOI:** 10.1002/anie.202111462

**Published:** 2021-11-26

**Authors:** Robin N. Perutz, Sylviane Sabo‐Etienne, Andrew S. Weller

**Affiliations:** ^1^ Department of Chemistry University of York York YO10 5DD UK; ^2^ CNRS LCC (Laboratoire de Chimie de Coordination) 205 route de Narbonne, BP 44099 F-31077 Toulouse Cedex 4 France

**Keywords:** agostic interactions, homogeneous catalysis, metathesis, organometallic reaction mechanisms, sigma-bond complexes

## Abstract

In 2007 two of us defined the σ‐Complex Assisted Metathesis mechanism (Perutz and Sabo‐Etienne, *Angew. Chem. Int. Ed*. **2007**, *46*, 2578–2592), that is, the σ‐CAM concept. This new approach to reaction mechanisms brought together metathesis reactions involving the formation of a variety of metal–element bonds through partner‐interchange of σ‐bond complexes. The key concept that defines a σ‐CAM process is a *single* transition state for metathesis that is connected by two intermediates that are σ‐bond complexes while the oxidation state of the metal remains constant in precursor, intermediates and product. This mechanism is appropriate in situations where σ‐bond complexes have been isolated or computed as well‐defined minima. Unlike several other mechanisms, it does not define the nature of the transition state. In this review, we highlight advances in the characterization and dynamic rearrangements of σ‐bond complexes, most notably alkane and zincane complexes, but also different geometries of silane and borane complexes. We set out a selection of catalytic and stoichiometric examples of the σ‐CAM mechanism that are supported by strong experimental and/or computational evidence. We then draw on these examples to demonstrate that the scope of the σ‐CAM mechanism has expanded to classes of reaction not envisaged in 2007 (additional σ‐bond ligands, agostic complexes, sp^2^‐carbon, surfaces). Finally, we provide a critical comparison to alternative mechanisms for metathesis of metal–element bonds.

## Graphical abstract

This review highlights advances in the characterization and dynamic rearrangements of σ‐bond complexes, most notably alkane and zincane complexes, but also different geometries of silane and borane complexes. We set out a selection of catalytic and stoichiometric examples of the σ‐complex assisted metathesis mechanism that are supported by strong experimental and/or computational evidence.

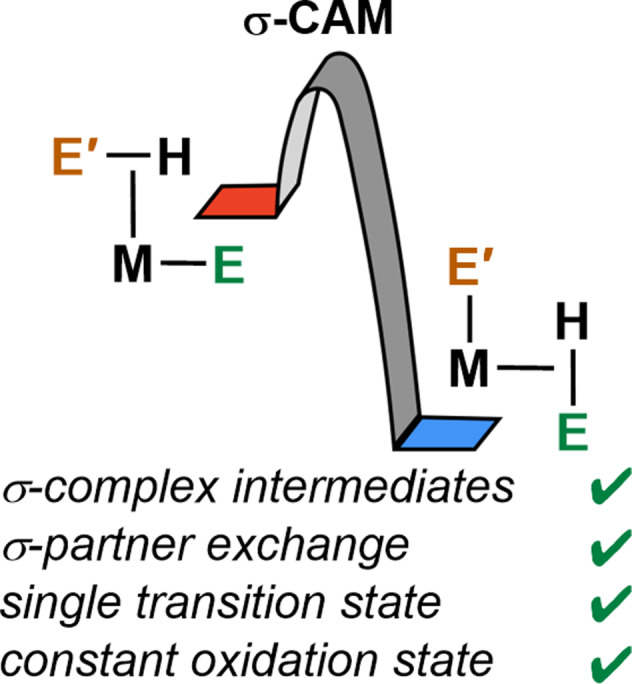

## 1 Introduction

**Scheme 1:**
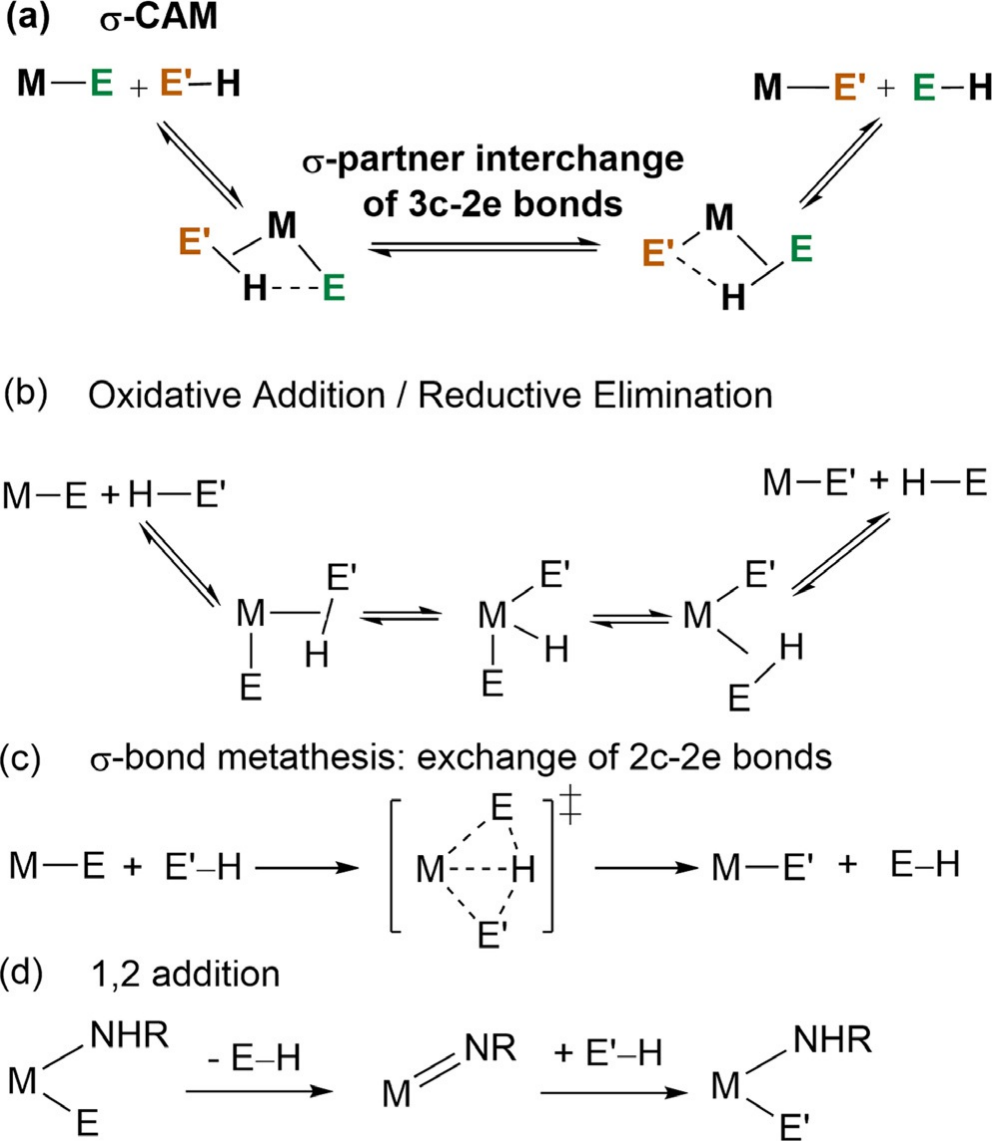
The σ‐CAM mechanism and three other mechanisms for metathesis at transition metal centers (E=H, C, Si, B).

In 2007 two of us published a review entitled “The σ‐CAM mechanism: σ‐complexes as the basis of σ‐bond metathesis at late‐transition‐metal centers”.^[1]^ The principle behind the proposed σ‐CAM (σ‐Complex Assisted Metathesis) mechanism is that σ‐bond complexes can interchange the partners that form the σ‐bond(s) donating to the metal. This interchange could lead to metathesis at constant oxidation state (Scheme 1 a). We proposed that such a mechanism would compete with oxidative addition/reductive elimination mechanisms (Scheme 1 b) in situations where the σ‐bond complexes acted as intermediates both preceding and following a single transition state that interchanged partners. Evidence for the existence of such σ‐complex intermediates may come from their direct spectroscopic observation or even crystallographic characterization. They are also often identified using computational methods, when their existence is fleeting or equilibrium concentrations are low in an overall reaction manifold. There was also a contrast with the standard σ‐bond metathesis mechanism of *d* ^0^ complexes (Scheme 1 c), because that did not require σ‐bond complexes as (potentially) observable intermediates.^[2]^ Similarly, 1,2‐addition (Scheme 1 d) is another transformation that breaks an E−H bond but does not require σ‐bond complexes as intermediates.

The underlying concept of a σ‐CAM process is an overall metathesis reaction that facilitates the replacement of one covalently, 2c–2e, metal‐bonded ligand by another [Eq. (1)]. For example, an alkyl ligand is replaced by a silyl ligand or vice versa. The entering reagent in this case is a silane and the co‐product is an alkane alongside the required metal silyl complex. This reaction could be used in synthesis where the new M−E′ complex is the main output, or as part of a larger catalytic manifold yielding the co‐product, E−H. 
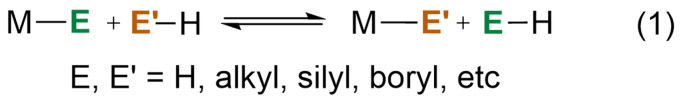


In the first step of the σ‐CAM mechanism, E′−H coordinates to the metal center to form a 3c–2e σ‐bond complex. This precursor σ‐bond complex undergoes a dynamic rearrangement to a new 3c–2e σ‐bond complex with coordinated E−H, and finally the co‐product E−H leaves generating the M−E′ product (Scheme 1 a). In by far the commonest version, hydrogen is exchanged between the σ‐bond partners (Scheme 1 a), but a less frequent alternative with E′ occupying the central position is discussed in section 3.4.

The overall reaction can be productive (E≠E′) or degenerate (E=E′). Common to both situations are three key features of the mechanism: (1) that a vacant site is required for the initial coordination of E′−H, (2) that two successive σ‐bond complexes are formed as reaction intermediates or isolable species, (3) the oxidation state of the metal center in the precursor, intermediates and product remains constant throughout the process. Unlike some other mechanisms, it does not specify the nature or oxidation state of the transition state (TS) between the two σ‐bond complexes, which can involve varying degrees of bonding between E, H and E′. However, importantly, a single TS should link the σ‐complexes which interchange partners.

The σ‐bond complexes may be detected by experiment or by computational methods. The formation of σ‐bond complexes depends on synergic bonding in which back‐bonding from the metal to the ligand is significant, although not dominant. Consequently, σ‐bond complexes require the presence of *d*‐electrons and are observed most commonly in *d* ^6^ and *d* ^8^ electron configurations.

The concept requires metathesis (i.e. conversion of M−E to M−E′) and not just dynamic interchange between two σ‐bond complexes. The σ‐bond metathesis mechanism commonly (but not exclusively) observed for *d* ^0^ configurations shares the feature of constant oxidation state but no σ‐bond complexes have been observed experimentally as intermediates. Instead, a kite‐shaped 4‐center TS is formed directly. These comparisons will be developed in sections 4 and 5.

In this review, we return to the σ‐CAM mechanism and examine a range of examples from many authors which have offered strong evidence in favor of the mechanism (Section 3). We also examine several different extensions of the principle and compare the σ‐CAM mechanism to other competing mechanisms (Sections 4,5). Before this, we outline advances in σ‐bond complex synthesis and characterization since the 2007 review, to provide context for the discussion of mechanism.

## 2 Major advances in structural variety of σ‐bond ligands and σ‐bond complexes

**Scheme 2:**
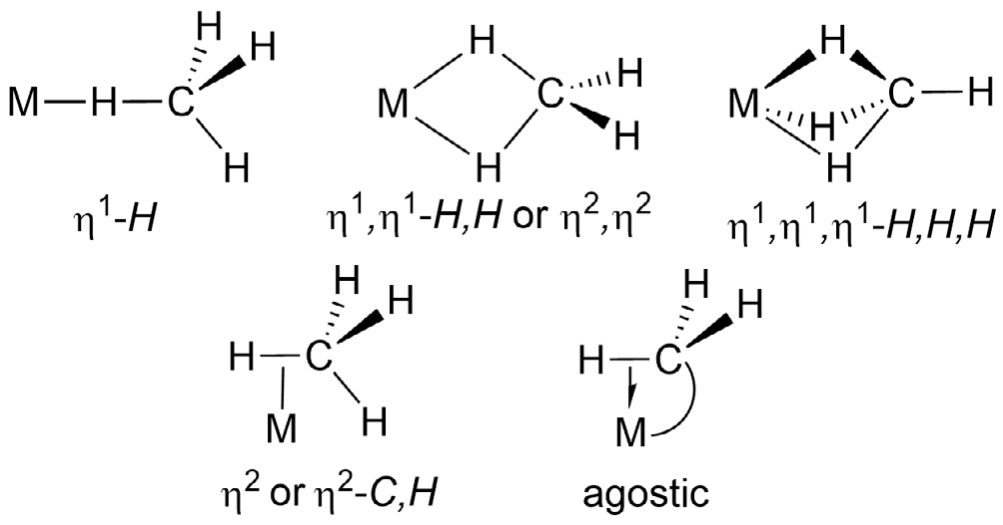
Possible coordination modes of methane as σ‐bond complexes and comparison to agostic interaction with corresponding nomenclature.

Traditional ligands for transition metals are bonded via donation of an electron pair either as 2c–2e (dative) covalent bond or by a π‐bond. It is also possible for a simple σ‐bond between a pair of atoms to act as donor to a metal center in a 3c–2e interaction. The resulting complexes are termed σ‐bond complexes, or simply σ‐complexes.[^3^, ^4^] The prototypical σ‐bond ligand is H_2_, first recognized by Kubas,^[5]^ but established examples can be found with alkanes, silanes, boranes and germanes. They typically exhibit η^1^‐ or η^2^‐*E‐H* geometries (E=H, C, B, Si, Ge) and are unsupported by other bonds to the metal, that is, they are intermolecular complexes. Other possible geometries are given in Scheme 2 in the context of alkanes together with definitions of our nomenclature.[^6^, ^7^, ^8^] The most appropriate nomenclature in systems with the potential for 4‐center interactions (middle of top row of Scheme 2) depends on detailed analysis of bonding^[9]^ which is beyond the scope of this review. This Scheme also illustrates the close relation to agostic complexes in which the M⋅⋅⋅H−C σ‐bond interaction is supported by another bond in an intramolecular chelate.^[10]^ Many authors have extended the agostic concept to other elements, most frequently silicon and boron; we specify the elements concerned if not C−H.[^11^, ^12^, ^13^, ^14^] When a complex contains both σ‐bond ligand(s) and either hydride or dihydrogen ligands, additional secondary interactions often occur revealing themselves by shorter internuclear distances than would otherwise be expected. In the specific case of Si⋅⋅⋅H, they are known as SISHA interactions (Secondary Interactions between Silicon and Hydrogen Atoms).

**Scheme 3:**
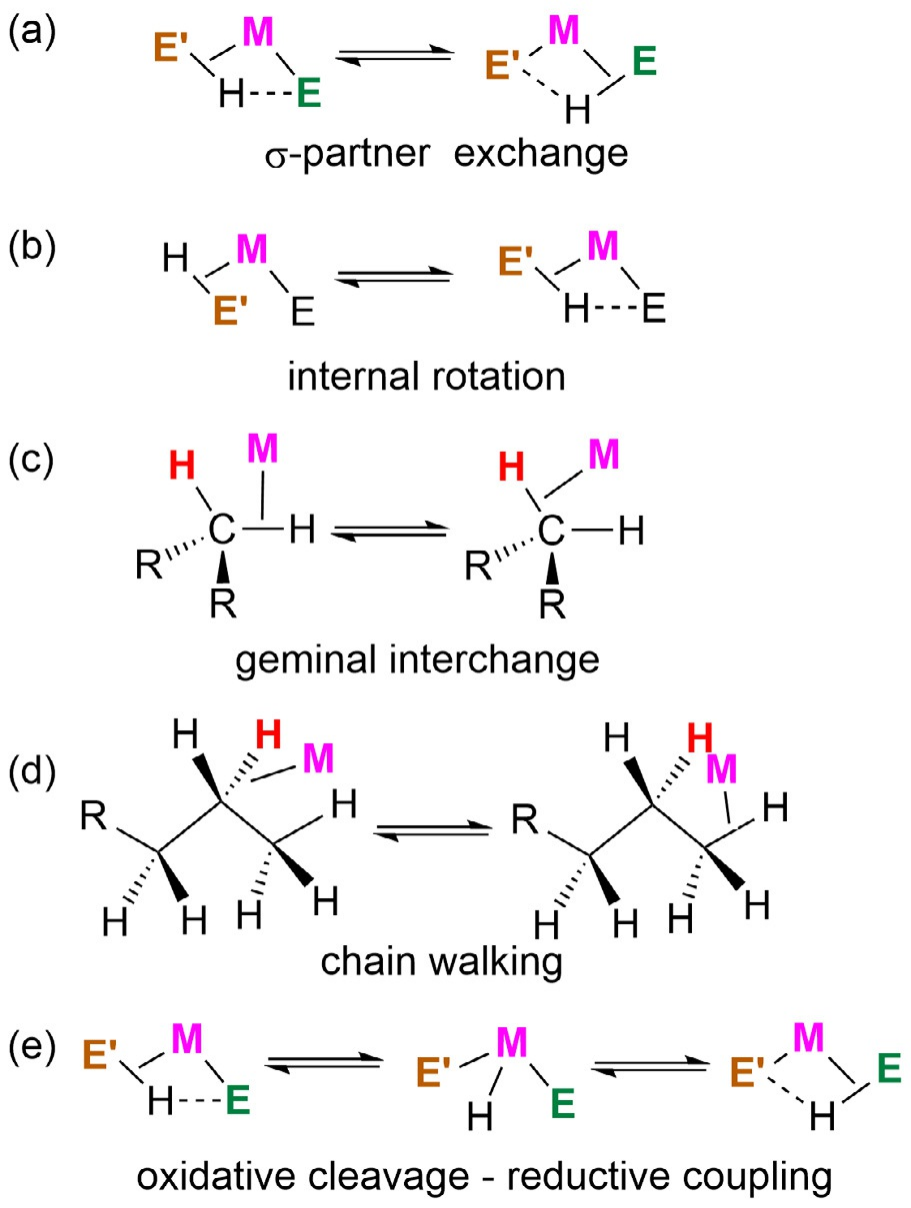
Dynamic processes of σ‐complexes.

Dynamic NMR experiments often reveal the fluxional processes that occur as the component nuclei of the σ‐ligand undergo interchange with their neighbors. Since the σ‐CAM mechanism demands the lengthening of the coordinated σ‐bond and the shortening of the distance to a neighboring ligand (Scheme 1 a), it is closely associated with the dynamic interchange of σ‐partners at constant oxidation state (Scheme 3 a). Such interchange may be assisted by the secondary interactions mentioned above. Internal rotation may also be required in some σ‐CAM mechanisms (Scheme 3 b). Geminal exchange and chain‐walking (Scheme 3 c,d) are related, dynamic processes that can occur in σ‐bond complexes but are not required in the σ‐CAM mechanism. This σ‐partner interchange also contrasts with the oxidative cleavage–reductive coupling mechanism that requires an intermediate of higher oxidation state (Scheme 3 e).

### 2.1 H−H σ‐bond complexes

Molecular dihydrogen complexes are now recognized to exist with a wide range of H−H distances (0.8–1.3 Å).[^5^, ^15^] Although H−H distances can be measured by single‐crystal X‐ray diffraction and estimated by NMR methods, the gold standard remains single‐crystal neutron diffraction structures.^[16]^ An example of a well‐characterized dihydrogen complex whose structure has been determined by single‐crystal neutron diffraction is Ru(H)_2_(H_2_)_2_(PCyp_3_)_2_ (Cyp=cyclopentyl, C_5_H_9_) in which the dihydrogen and hydride ligands occupy the equatorial belt around Ru, with the phosphine ligands axial.^[17]^ Access via H/D exchange to the deuterium isotopologue, Ru(D)_2_(D_2_)_2_(PCyp_3_)_2_, allows for the exploitation of the very different scattering cross‐sections for hydrogen and deuterium to trace the isotopic exchange. The structure also provides Ru−D and D−D bond lengths with estimated standard deviations of 0.002 Å or less (see section 3.1 for a discussion of the exchange mechanism).^[16]^ Even with neutron diffraction, however, the distinctions between hydride and dihydrogen can sometimes be blurred due to disorder^[18]^ or nuclear motion on a flat potential energy surface.^[19]^ Dihydrogen complexes have also been identified at metal nodes in metal‐organic framework materials using powder neutron diffraction and IR spectroscopy,^[20]^ and on metal nanoparticle surfaces using ^2^H solid‐state NMR techniques.^[21]^

### 2.2 C−H σ‐bond complexes

**Figure 1:**
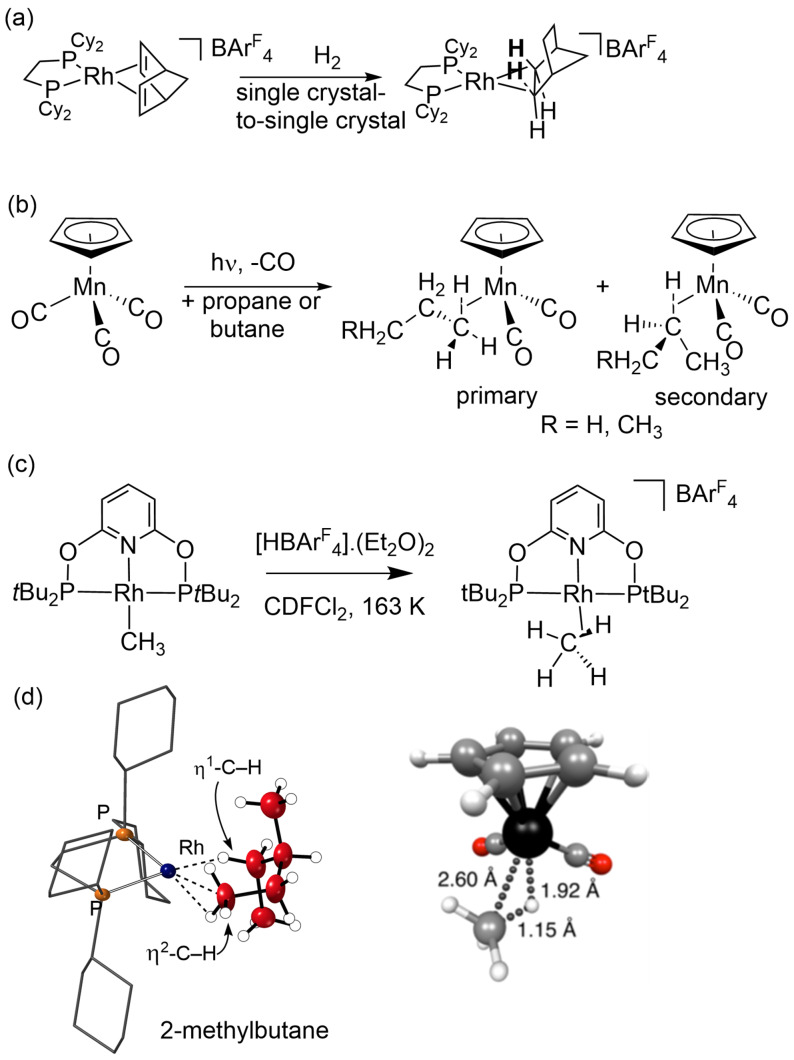
(a) Synthesis of [Rh(Cy_2_PCH_2_CH_2_PCy_2_)(η^2^,η^2^‐norbornane)][BAr^F^_4_]; (b) photochemical synthesis of manganese propane and butane complexes; (c) synthesis of rhodium methane complex by protonation; (d) experimental structure of cation [Rh(Cy_2_PCH_2_CH_2_PCy_2_)(η^2^,η^1^‐2‐methylbutane)]^+^ showing coordination at rhodium by the alkane and calculated structure of CpRe(CO)_2_(η^2^‐CH_4_) at CCSD(T)/def2‐QZVPP level.^[48]^

Major advances in the understanding of alkane σ‐complexes have been made since our review in 2007.[^8^, ^22^, ^23^, ^24^] Most notably, several rhodium complexes and one cobalt complex have been characterized by single crystal X‐ray diffraction, using single‐crystal to single‐crystal reactivity of molecular alkene precursors by simple addition of H_2_, providing the long sought geometric proof of structure (Figure 1 a).[^25^, ^26^, ^27^, ^28^, ^29^, ^30^, ^31^] The majority of these complexes contain an alkane ligand coordinated by two C−H bonds on different carbon atoms to the metal, each in a M(η^2^‐C−H) mode (Figure 1). M(η^1^‐C−H) coordination modes are also reported depending on the identity of the metal/ligand/alkane. Many of these complexes can be observed at room temperature, a consequence of the stabilizing non‐covalent interactions provided by the secondary anion microenvironment in the solid state.[^31^, ^32^] Isotope H/D exchange at the bound alkane ligand using D_2_ allows for remarkable selectivity in such processes as determined using single crystal neutron diffraction techniques.^[33]^ Alkane complexes have also been synthesized in solution by low‐temperature photolysis of metal carbonyl, or metal dinitrogen, precursors and by protonation of metal methyl complexes (Figure 1 b). Low temperature ^1^H NMR spectroscopy in solution has revealed the isotopic perturbation of resonance for the η^2^‐C−H bond of the partially deuterated isomers. This effect demonstrates that rapid and reversible exchange processes are occurring between C−H (C−D) bonds that can interact with the metal center.^[34]^ Additionally, the corresponding ^13^C resonance of the alkane ligand can lie at an exceptionally high field and exhibits a reduced C−H coupling constant when compared to the free alkane (Figure 1 b,c).[^34^, ^35^, ^36^, ^37^, ^38^, ^39^, ^40^, ^41^, ^42^]

Metal centers that have been shown to engage in σ‐alkane complex formation now include W, Mn, Re, and Rh and contain a variety of supporting ligands. This improved characterization has been accompanied by quantitative measurements of reactivity including dynamic exchange processes in solution[^34^, ^35^, ^36^, ^37^, ^38^, ^39^, ^40^, ^43^] revealed by time‐resolved infrared spectroscopy,[^34^, ^40^, ^44^, ^45^] or by time‐resolved EXAFS.^[46]^ Dynamic exchange in the solid‐state has been observed using low temperature solid‐state NMR spectroscopy.[^26^, ^28^, ^33^] The level of theory in computational studies has also improved considerably so that reliable comparisons may be made of the interactions of different alkanes with metal centers using isolated molecule (gas‐phase) calculations.[^47^, ^48^, ^49^] For example, the methane complex CpRe(CO)_2_(η^2^‐CH_4_) has been analyzed using coupled‐cluster methods by two groups (Figure 1 d).[^48^, ^49^] These two papers agree broadly on binding energies (62.0 kJ mol^−1^ and 70.0 kJ mol^−1^) and on the importance of dispersion, but disagree on the magnitude of the dispersion contribution. Neither of them account for the solvent contribution to dispersion. In the solid‐state, periodic DFT methodologies can be used to interrogate binding, stability and reactivity in σ‐alkane complexes.^[50]^ The ability to stabilize σ‐alkane complexes at room temperature using single‐crystal methodologies means that onward reactivity of the M⋅⋅⋅H−C interaction becomes kinetically accessible. Reactions have been studied that connect σ‐alkane complexes with the products of C−H activation: for example, selective H/D exchange and acceptorless alkane dehydrogenation.[^26^, ^31^, ^33^]

**Figure 2:**
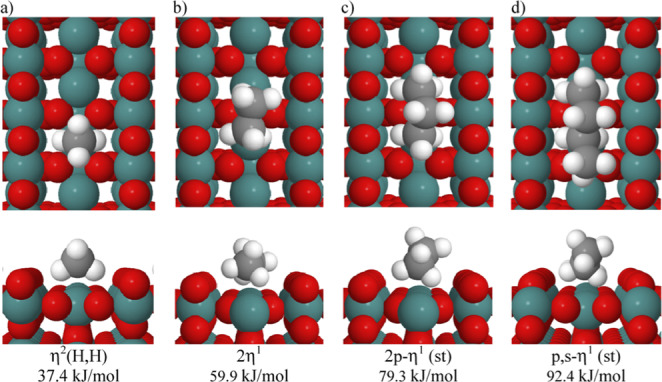
Preferred configurations and binding energies of (a) methane (b) ethane, (c) propane, (d) *n*‐butane adsorbed on a RuO_2_(110) surface as computed by DFT‐D3 methodologies. Reproduced with permission from ref. [53].

σ‐Alkane complexes have also been directly characterized on metal oxide surfaces, such as RuO_2_ or PdO, at low temperatures (e.g. 90 K) using a combination of temperature‐programmed desorption, surface IR spectroscopy and DFT techniques.^[51]^ Reassuringly, these M⋅⋅⋅H‐C interactions are broadly similar to those observed and calculated for molecular species, albeit now with the possibility of interaction with multiple surface metal sites for alkanes larger than methane (Figure 2). The interaction of cyclic alkanes with small (Ru_13_) nanoparticles has been studied using DFT computational methods to understand empirically observed H/D exchange processes. These calculations indicate the formation of σ‐alkane complexes on the nanoparticle surface prior to C−H bond cleavage.^[52]^ We return to these systems in our discussion of the σ‐CAM mechanism later (Section 3.5).

### 2.3 Si−H σ‐bond complexes

A wide variety of silane and disilane complexes as well as SiH‐agostic complexes have been prepared.[^11^, ^54^] It has now been demonstrated that a simple hydrosilane (Et_3_SiH) can bond in an η^1^‐geometry (**1**) as well as an η^2^‐geometry, paralleling the behavior of alkanes mentioned above. DFT calculations (B3LYP/LANL2DZ/6‐311G**) suggest less Ir dπ to SiH σ* backbonding in the η^1^‐SiH complex than with a conventional η^2^‐silane.^[55]^ An example of an η^1^‐SiH complex undergoing onward reactivity comes from a cyclometalated platinum complex with a supporting σ‐bond silane ligand that reacts to form a conventional Pt−Si bond and open the cyclometalated ring.^[56]^ 
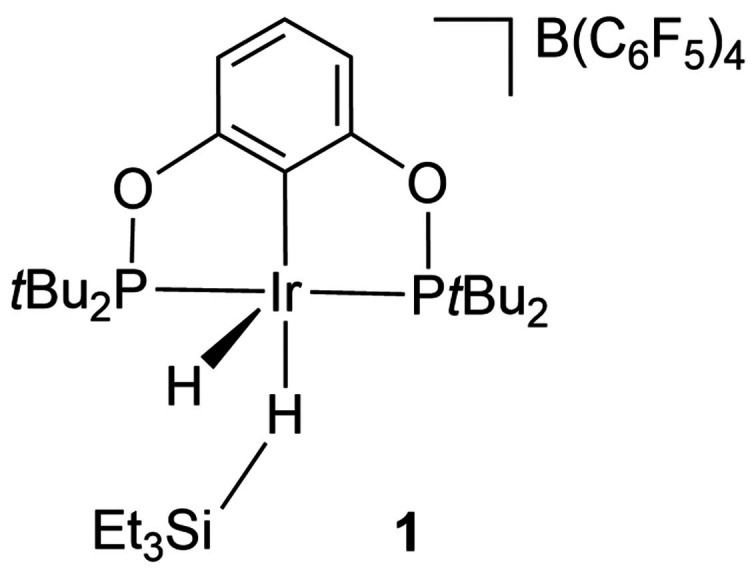


**Figure 3:**
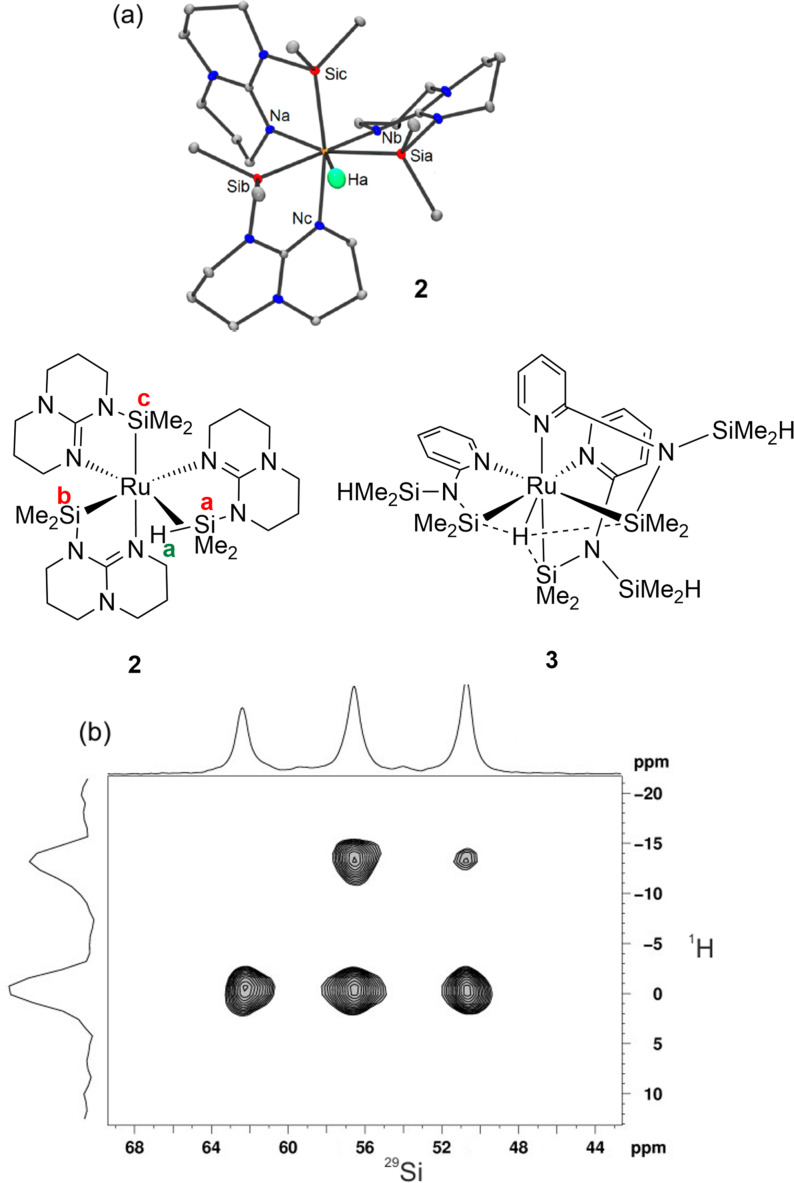
(a) Complexes **2** and **3** with neutron diffraction structure of **2**; (b) solid state ^1^H‐^29^Si HETCOR NMR spectrum of **2**. Adapted from ref. [57].

The ruthenium silazane complex **2** which contains a metal‐hydrogen and three metal‐silicon bonds represents an example of structural characterization of an η^2^‐SiH complex. It has been studied by neutron diffraction, solution and solid state NMR and by DFT calculations (B3PW91).^[57]^ The structural evidence (Figure 3 a) shows that the distances from the three Si atoms to the single hydride are all different. One is described as a Si−H bond (Si^a^ 1.874(3) Å), the next as a Si⋅⋅⋅H or SISHA interaction (Si^B^ 2.099(3) Å), and the third as non‐bonding (Si^c^ 3.032(3) Å, quoting neutron diffraction distances). The Ru−Si^a^ and Ru−Si^b^ distances are essentially equal while the Ru‐Si^c^ distance is slightly shorter. In solution, the Si nuclei are indistinguishable by NMR at all temperatures accessed, but the solid‐state Si−H HETCOR NMR spectrum (Figure 3 b) clearly shows the Si nuclei as inequivalent with two of them coupled to the hydride. In contrast, the related complex **3** shows equal Ru−Si distances and equivalent Si nuclei even in the solid‐state NMR spectrum; this species is described as a Ru^IV^(SiMe_2_R)_3_H complex stabilized by SISHA interactions between the hydride and all three Si atoms.^[58]^

**Scheme 4:**
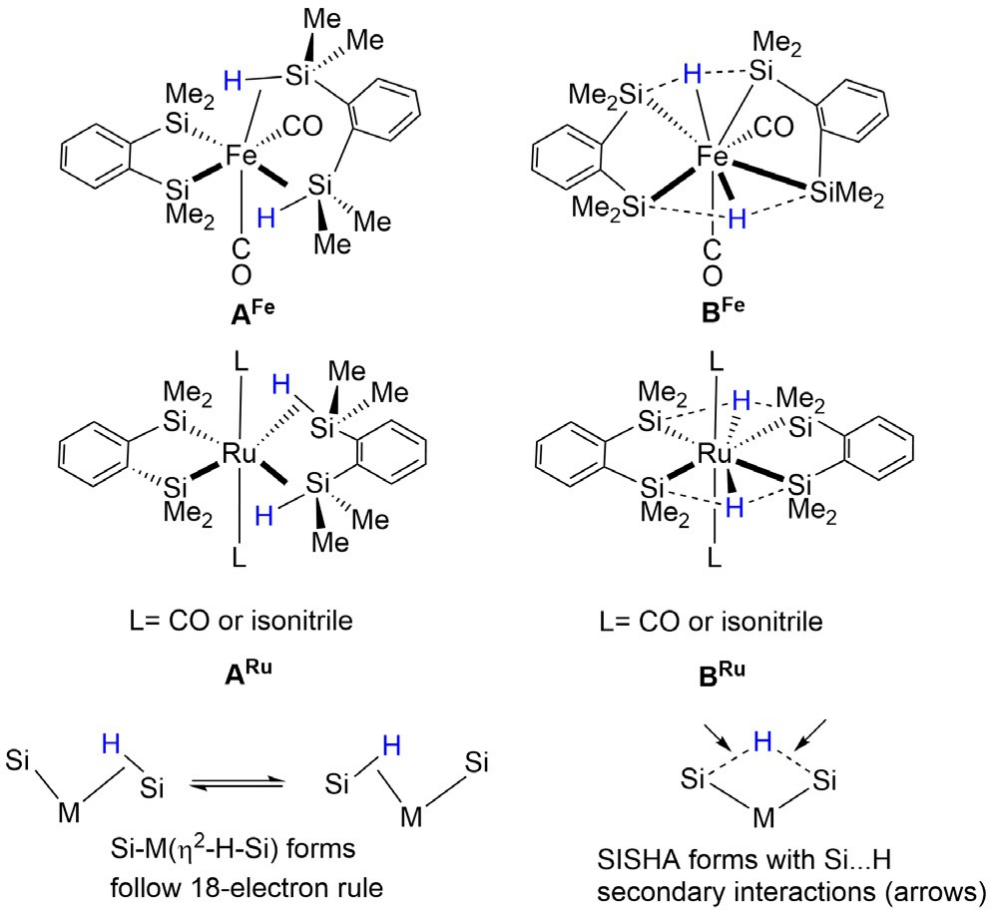
η^2^‐Silane and SISHA forms of iron and ruthenium complexes of 1,2‐bis(dimethylsilyl)benzene, adapted from ref. [61].

Complexes of iron and ruthenium formed by reaction of precursors with 1,2‐bis(dimethylsilyl)benzene formally contain one SiMe_2_(C_6_H_4_)Me_2_Si unit bound to the metal by conventional 2c‐2e σ‐bonds, and one H‐SiMe_2_(C_6_H_4_)Me_2_Si‐H unit bound by η^2^‐Si‐H interactions (**A^Fe^, A^Ru^**). However, there is an alternative formulation in which each hydrogen is bound as a hydride to the metal and engaged in secondary SISHA bonding to two silicons (**B^Fe^, B^Ru^**). The spectroscopic and crystallographic data support the M‐H + SISHA formulation (Scheme 4). Thus the metals are coordinated by 2 L ligands (carbonyl or isonitrile), 4 silicon atoms and 2 hydrides. These hydrides lie midway between pairs of silicon atoms and undergo secondary interactions with them rendering the σ‐bond complex description inappropriate.[^59^, ^60^, ^61^]

**Figure 4:**
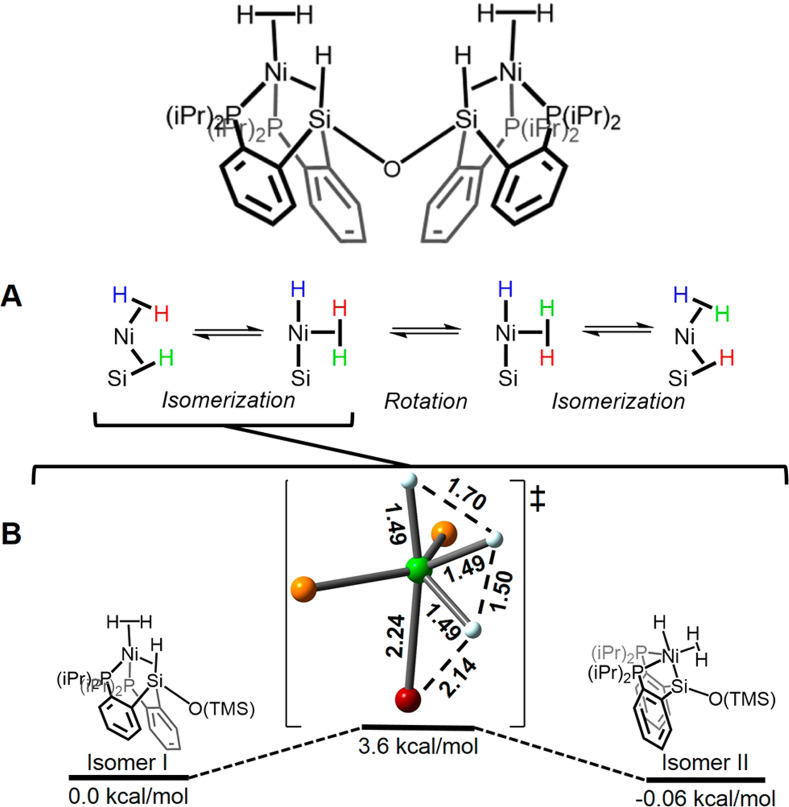
Above: Ni^0^_2_ complex with η^2^‐H_2_ and η^2^‐SiH‐coordinated P_2_SiOSiP_2_ ligands. (A) pathway for exchange of H atoms between the η^2^‐H_2_ and η^2^‐SiH ligands. (B) transition state for interconversion of isomers located by DFT (Ni green; P yellow; Si red; H white). Adapted from ref. [62].

A Ni_2_ complex with a dinucleating P_2_SiOSiP_2_ ligand provides an intriguing example of dynamic exchange between dihydrogen and silane ligands that is the key step in catalytic silane deuteration. The square‐planar precursor contains two Ni^II^ units, each with a hydride, a silyl and two phosphine ligands bridged by the SiOSi group. On reaction with H_2_, this complex reacts to generate first one, and subsequently two Ni^0^ units, each with a dihydrogen, an η^2^‐SiH and two phosphine ligands.^[62]^ NMR spectra show that the SiH and H_2_ groups undergo dynamic exchange at room temperature but coalesce at −90° C. The proposed exchange mechanism (BP86, 6‐31G(d)) is presented in Figure 4.

The ability of mono‐silanes to bridge two metals has been illustrated previously.^[63]^ More recently, an intriguing example of such behavior was reported for a Ni_2_ complex bridged by H_2_SiR_2_ (R=Ph, Et) **4** in which the hydrogen atoms lie midway between Ni and Si and the H‐Si‐H angle is opened to 156(3)°.^[64]^ Calculations suggest that, due to the doubly reduced naphthyridine‐diimine ligand, these complexes are best considered as Ni^I^‐Ni^I^ species on the continuum between a σ‐complex and final double Si‐H oxidative addition. Most unusually for a silane complex, the NMR spectra reveal that there is a triplet excited state slightly above the singlet ground state. 
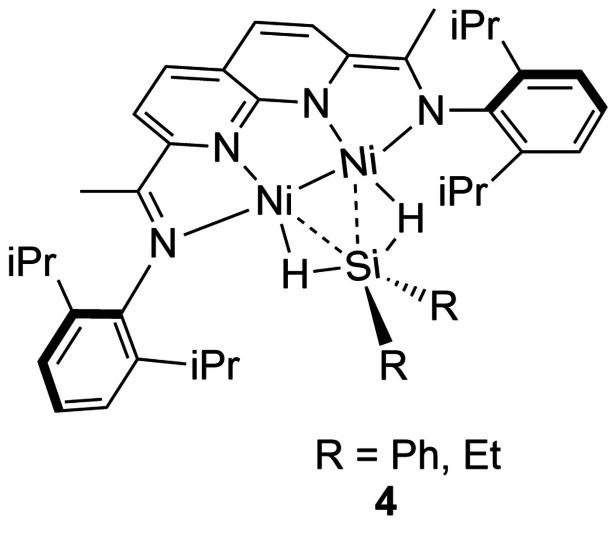


**Figure 5:**
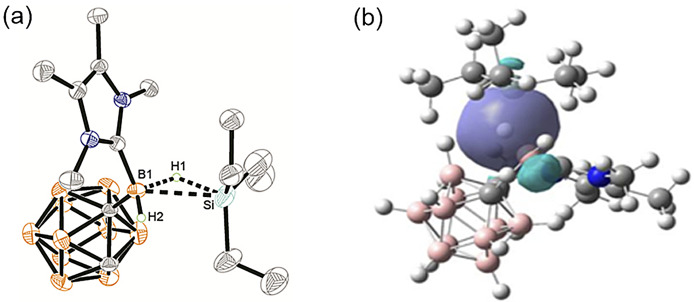
(a) Molecular structure of [IMe_4_‐(Cb)(μ‐H)(HSiEt_3_)][B(C_6_F_5_)_4_] (for abbreviations, see text); (b) NBO analysis of the B⋅⋅⋅H−Si interaction. Reproduced with permission from ref. [65].

An intriguing main group σ‐bond complex, [IMe_4_‐(Cb)(μ‐H)(HSiEt_3_)][B(C_6_F_5_)_4_], (IMe_4_=1,3,4,5‐tetramethylimidazol‐2‐ylidene, Cb=1,2‐dicarba‐closo‐dodecaborane) has been described in which a silane (HSiEt_3_) is proposed to engage with the empty *p*‐orbital of a cationic borenium center in a B(η^2^‐Si‐H) σ‐interaction (Figure 5).^[65]^ Other complexes have been reported where Si−H⋅⋅⋅B^[66]^ or C−H⋅⋅⋅Si bonds^[67]^ are invoked. It will be interesting to see if σ‐CAM is extended to reactions involving only main group elements.

### 2.4 B−H σ‐bond complexes

**Scheme 5:**
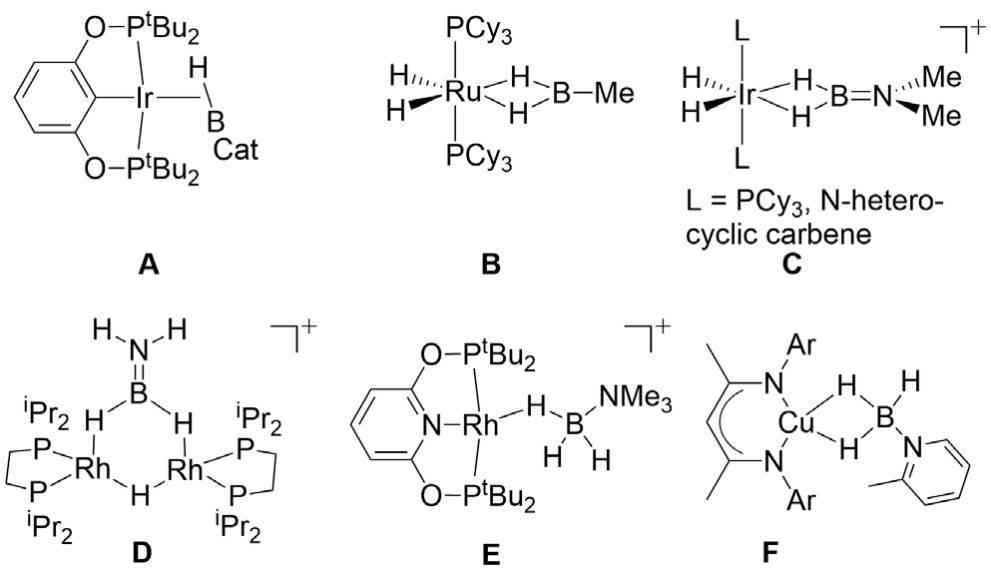
Examples of bonding modes in σ‐bond complexes of 3‐ and 4‐coordinate boranes.

Since the initial review article in 2007 there has been significant interest in the coordination chemistry, and onward reactivity, of B−H σ‐bond complexes. Such complexes play a role in: (a) the construction of C−B bonds via C−H activation strategies^[68]^ using 3‐coordinate boranes such as HBCat or HBpin (Cat=catecholate, pin=pinacolate); (b) the catalytic removal of H_2_ from amine‐boranes, prototypically H_3_B⋅NR_3_ (R=alkyl or H), for proposed hydrogen storage applications[^69^, ^70^, ^71^, ^72^, ^73^] and for the synthesis of new B‐N main chain containing polymeric materials.^[74]^ Related to these studies, the coordination chemistry and reactivity of dihydrido‐boranes (H_2_BR), amino‐boranes (H_2_B=NR_2_) and phosphine‐boranes H_3_B‐PR_3_ has been developed. A wide range of σ‐bonding coordination modes are expressed in such complexes, and selected examples (Scheme 5) of 3‐coordinate (**A**–**D**) and 4‐coordinate (**E**–**F**) borane species include: M(η^2^‐B‐H), **A**;^[75]^ M(η^2^,η^2^‐BH_2_), **B** ^[76]^ and **C**;[^77^, ^78^] M_2_(μ,η^2^,η^2^‐BH_2_), **D**;^[79]^ M(η^1^‐BH_3_), **E**;^[80]^ M(η^2^,η^2^‐BH_3_), **F**.^[81]^ In addition to these mono‐boron species, σ‐bond complexes from ligands that contain more than one boron (including boron clusters) are being actively investigated.[^12^, ^82^]

Computational studies on these complexes demonstrate that the bonding between the metal and the borane is best described as arising from donation from a B−H bonding orbital. For 3‐coordinate boranes back‐donation into low lying B−H σ* orbitals, or an unoccupied *p*‐orbital, is also significant.^[77]^ In 4‐coordinate boranes, σ‐donation to the metal dominates and there is little evidence for back‐bonding since the σ* B−H orbital lies at high energy.

**Figure 6:**
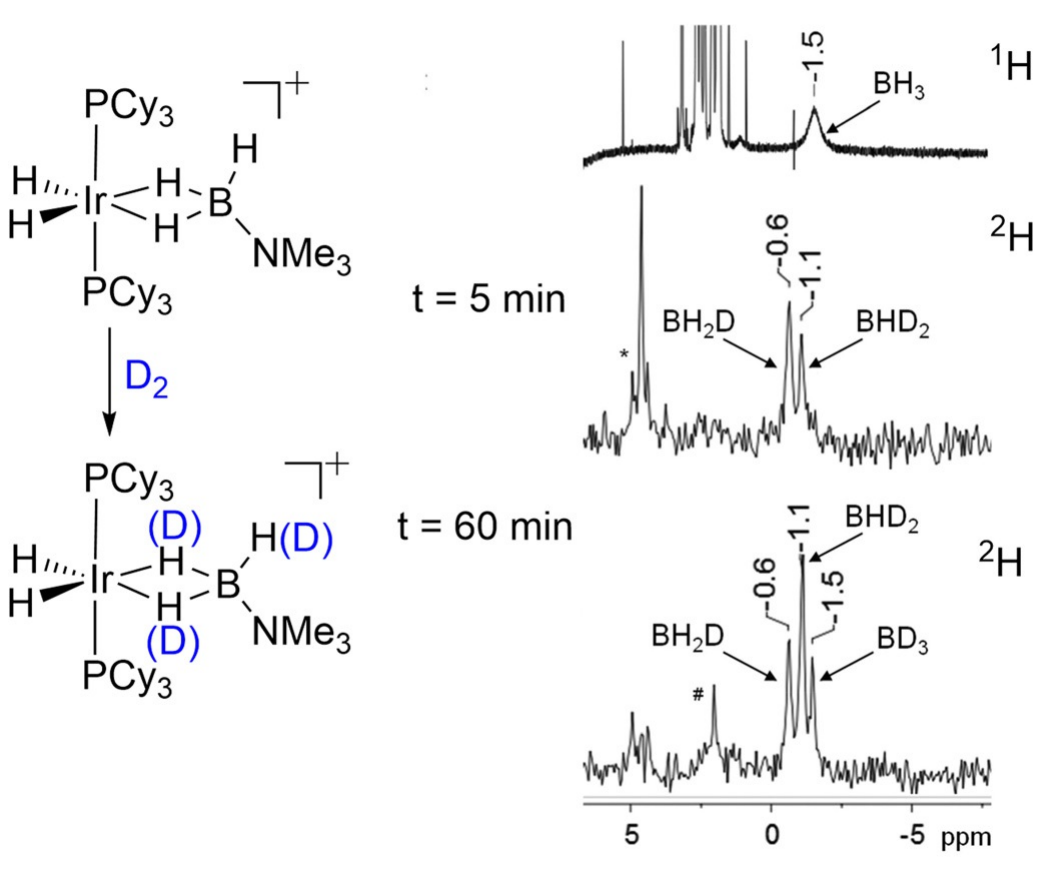
Partial H/D exchange under D_2_ and the observation of isotopic perturbation of equilibrium in [Ir(PCy_3_)_2_(H)_2_(η^2^η^2^‐H_3_B⋅NMe_3_)][BAr^F^_4_] using ^1^H and ^2^H NMR spectroscopy. Time=0 (^1^H NMR spectrum), Time=5, 60 min (^2^H NMR spectra). The signals close to *δ* 5 are due to H_2_, HD and D_2_. Reproduced with permission in part from ref. [84].

Boron‐hydrogen bonds are rather hydridic due to the electronegativity difference between boron and hydrogen, and this, in turn, is a contributor to the greater stability in solution of σ‐borane complexes compared to their σ‐alkane counterparts. Consequently, detailed NMR characterization is possible at room temperature and single crystals may be produced using traditional solution techniques. This difference is illustrated by a comparison of the stabilities of two closely related complexes: Mn(η^5^‐C_5_H_5_)(CO)(propane)^[34]^ and Mn(η^5^‐C_5_H_5_)(CO)(H_3_B⋅NMe_3_).^[83]^ The former is only observed at 134 K in liquid propane, while the latter is stable at room temperature and can be recrystallized to allow for a structural characterization. Like their isoelectronic alkane counterparts, amine borane ligands (H_3_B⋅NR_3_) are often highly fluxional, undergoing exchange of geminal M⋅⋅⋅H−B interactions, as well as H/D exchange at the B−H groups with D_2_. Figure 6 demonstrates that both of these process occur in [Ir(PCy_3_)_2_(H)_2_(η^2^,η^2^‐H_3_B⋅NMe_3_)][BAr^F^_4_], where partial substitution of B−H for B−D using D_2_ leads to a series of isotopologues with B(H_3−*x*_D_*x*_) (*x=*3‐0).^[84]^ This H/D exchange results in an example of isotopic perturbation of equilibrium of a σ‐borane complex detected in the resulting ^1^H and ^2^H NMR spectra. This phenomenon comes from the preference for B−D to adopt terminal rather than bridging positions^[85]^ when in fast exchange with B−H bonds on the NMR timescale. Consequently, there are downfield shifts of the BH_2_D signal (*δ*−0.6) and the BHD_2_ signal (*δ*−1.1) relative to the BD_3_ (*δ*−1.5) in the ^2^H NMR spectrum. The mechanism by which H/D exchange proceeds is discussed in the σ‐CAM section (Section 3.4).

**Figure 7:**
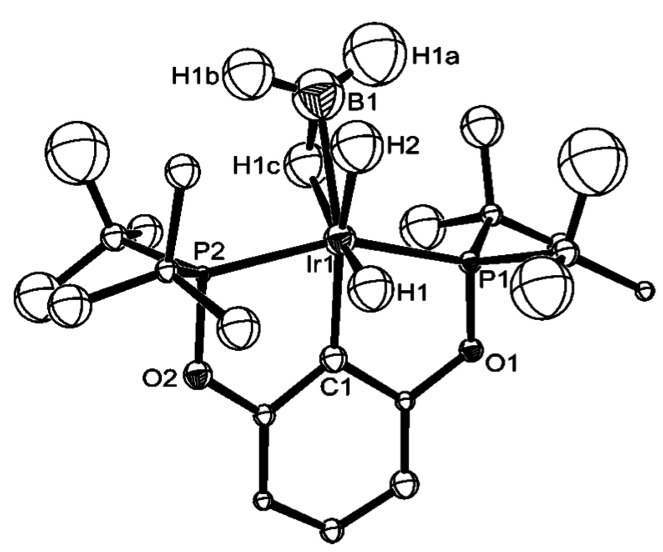
Molecular structure of Ir(^t^BuPOCOP)(H)_2_(η^2^‐HBH_2_) as determined by single‐crystal neutron diffraction. Reproduced with permission from ref. [75].

Finally, the structure of a σ‐bound BH_3_ ligand, Ir(^t^BuPOCOP)(H)_2_(η^2^‐HBH_2_) [^t^BuPOCOP=κ^3^‐C_6_H_3_‐1,3‐(OP^t^Bu_2_)_2_] has been characterized using single‐crystal neutron diffraction (Figure 7).^[75]^ Careful consideration of the bonding metrics points to a formulation as a σ‐bond complex of BH_3_ rather than a tetrahydridoborate complex with a very activated B−H bond. Thus, B1−H1c is lengthened compared to the non‐interacting terminal B−H bonds [1.45(5) Å versus 1.18(2) and 1.22(5) Å], consistent with σ‐coordination, and the distance to the proximal Ir‐hydride is too long to be considered a covalent bond [B1−H2, 1.74(5) Å].

### 2.5 E−H σ‐bond complexes (E=Al, Ga, Zn)

**Scheme 6:**
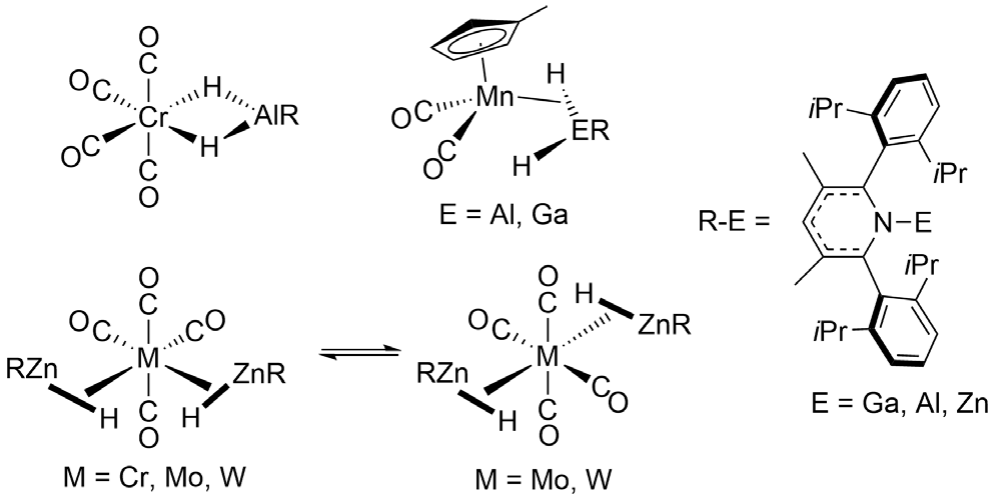
Transition metal σ‐bond complexes of η^1^,η^1^‐*H,H*‐AlH_2_, η^1^,η^1^‐*H,H*‐GaH_2_ and η^2^‐Zn‐H.

**Scheme 7:**
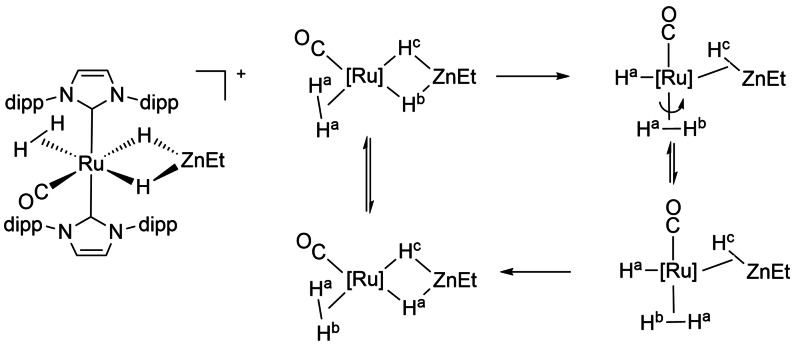
Ruthenium η^1^,η^1^‐*H,H* zincate cation and its exchange mechanism adapted from ref. [90].

New additions to the range of σ‐complexes derive from the chemistry of hydrides of main group metals of groups 12 and 13, when coordinated to transition metals; both η^2^‐*E,H* and η^1^,η^1^‐*H,H* forms have been reported for E=Al, Ga, Zn (Scheme 6).[^86^, ^87^, ^88^] A critical assessment provides parameters for deciding whether the σ‐alane, σ‐gallane, σ‐zincane formulation best represents the bonding situation.^[88]^ Two important criteria are (a) the formal shortness ratio, defined as the ratio of the M−M′ distance to the sum of the single bond radii of the transition metal M and the main group metal M′ and (b) the CO‐stretching frequency of metal carbonyl derivatives. For an example of a structurally characterized bis σ‐zincane complex, we consider Cr(CO)_4_(η^2^‐H−ZnR)_2_ (R=β‐diketiminate) in which the hydrogen atoms have been located using single‐crystal X‐ray diffraction. Notably, the two CrHZn units are coplanar (Scheme 6). The formal shortness ratio exceeds 1.0 as expected for a σ‐zincane complex and the low wavenumbers of ν(CO) support a formulation as Cr^0^. The alternative formulation as a dihydrogen complex was excluded in solution by NMR relaxation time measurements (definitely for Mo and W, less decisive for Cr). The Mo and W analogues exhibit two isomers that interconvert by an intramolecular mechanism.^[89]^ In a similar way to that found with boron, the η^2^‐*Zn,H* geometry of these neutral ligands is different from that of formally anionic dihydrozincate ligands that show a η^1^,η^1^‐*H,H* geometry as in the ruthenium complex in Scheme 7. This complex exhibits exchange between the dihydrogen ligand and the dihydrozincate hydrogens that proceeds via an η^2^‐ZnHEt ligand (Scheme 7).^[90]^ In further examples, the bis(η^2^‐zincane) complexes M(PCy_3_)(η^2^‐H−ZnR)_2_ (M=Pd, Pt) are formed by reaction of the β‐ketiminate zinc hydride RZnH (see Scheme 6 for ligand R) with M(PCy_3_)_2_.^[91]^ The Pt complex reacts with pentafluorobenzene to form Pt(C_6_F_5_)(PCy_3_)(η^1^,η^1^‐*H*,*H*‐H_2_ZnR) in which both hydrides interact with both Pt and Zn.^[91]^

**Figure 8:**
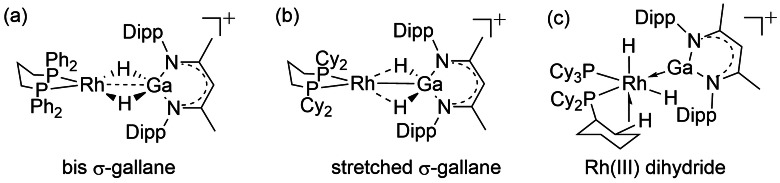
Structural snapshots of Ga−H bond activation at rhodium in [Rh(bisphosphine){H_2_Ga(NacNac)}][BAr^F^_4_] with three bisphosphines (a) dppp (Ph_2_PCH_2_CH_2_CH_2_PPh_2_); (b) dcypp (Cy_2_PCH_2_CH_2_CH_2_PCy_2_); (c) (PCy_3_)_2_. [BAr^F^_4_]^−^ anions not shown.^[92]^

The extent of Ga−H bond activation in [Rh(bisphosphine){H_2_Ga(NacNac)}][BAr^F^_4_] [NacNac = HC(MeCN(2,6‐^i^Pr_2_‐C_6_H_3_)_2_] can be systematically controlled by the combined effects exerted by bite angle of the chelating ligand and the steric bulk of ancillary R groups.[^92^, ^93^] This leads to structural snapshots of Ga−H σ‐bond activation at a metal center (Figure 8): from a bis‐σ‐bond complex (dppp), through stretched Ga−H bonds (dcypp) to a fully Ga−H activated Rh^III^ dihydride (PCy_3_) with a Ga^I^ L‐type ligand. Computational studies (BP86‐def2TZVP‐D3BJ) on the bis σ‐bond complex [Rh(dppp){H_2_Ga(NacNac)}][BAr^F^_4_] show the expected synergic bonding, with donation from the HOMO Ga−H bond to the LUMO of the cationic Rh‐fragment complemented by back donation from the metal into Ga−H σ* orbitals.

### 2.6 E−E σ‐bond complexes

Once considered exceedingly rare, σ‐bond complexes that involve E−E bonds (e.g. B, C, Si) have peppered the literature over the last 15 years.[^94^, ^95^] While still relatively uncommon, such interactions are now firmly established, and have been characterized by structural (single crystal X‐ray diffraction), spectroscopic (NMR) and computational (DFT/QTAIM) techniques. Selected examples are included here to highlight key advances, alongside the various descriptors that are used to identify E−E σ‐bond coordination.

**Scheme 8:**
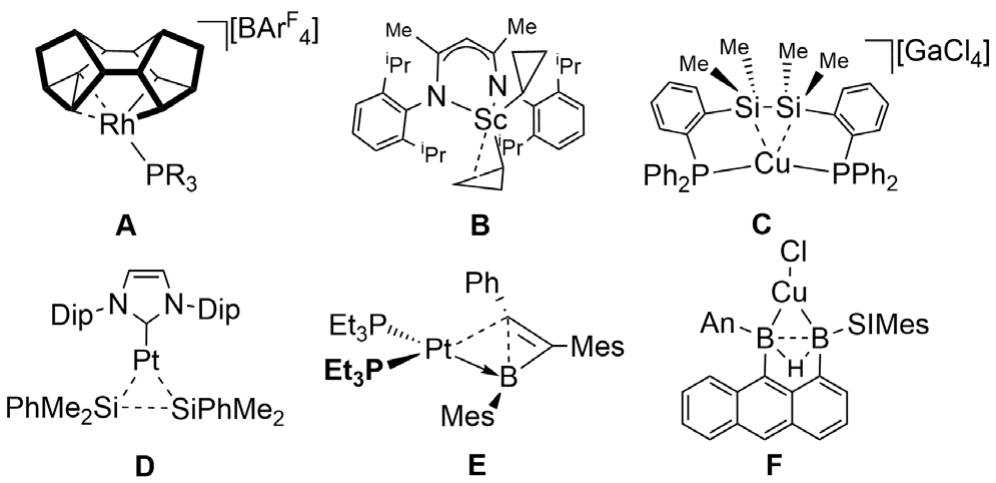
Examples of E−E′ σ‐bond complexes (E,E′=carbon or boron). An=9‐anthryl, SIMes=1,3‐bis(2,4,6‐trimethylphenyl)‐4,5‐ihydroimidazolidin‐2‐ylindene, Dip=2,6‐diisopropylphenyl.

The intramolecular coordination of C−C single bonds has been demonstrated in group 9 systems using a ligand derived from the saturated hydrocarbon Binor‐S, [Rh(PR_3_)(Binor‐S′)][BAr^F^_4_] (Binor‐*S*=1,2,4,5,6,8‐dimetheno‐s‐indacene, R=^i^Pr, Cy, Cyp), **A** (Scheme 8). In these Rh^III^ complexes, a metallocyclobutane unit is partnered with a cyclopropyl C−C agostic interaction with the metal from the Binor‐S derived ligand. This agostic C−C σ‐interaction results in a significant lengthening of the C−C bond compared with free derivatives of Binor‐S, the observation of Rh−C coupling in the low temperature ^13^C{^1^H} NMR spectra, and bond critical points between the C−C unit and the Rh as determined by both DFT/QTAIM (LDA/VWN/BP86‐6‐31G**) and experimental charge density studies.^[96]^ An Ir‐congener is also reported, that undergoes reversible C−C activation in single‐crystal to single‐crystal processes in the solid‐state.^[97]^ C−C agostic interactions have also been studied extensively in early transition metal systems that contain cyclopropyl ligands.^[98]^ In a recent example, Sc(L)(*c*‐C_3_H_5_)_2_ [L=N(2,6‐^i^Pr_2_‐C_6_H_3_)C(Me)CHC(Me)N(2,6‐^i^Pr_2_‐C_6_H_3_)], **B**,^[99]^ C−C agostic interactions between the cyclopropyl group and the metal center are signaled by lengthening of the C−C bond that closely approaches the metal, a reduced ^13^C−^13^C ^1^ *J* coupling constant, and NBO analysis (PBE0‐GD3‐BJ). A β‐agostic C−H bond in the cyclopropyl ring is also involved in donation to the metal center in some cases. Similar η^3^‐C−C−H agostic interactions have been mentioned as intermediates calculated in C−C and C−H oxidative cleavage processes at Rh^I^ centers.^[100]^ Intramolecular Si−Si σ‐bond interactions with Cu^[101]^ have also been described in which a Si−Si single bond is brought in close approach to a Cu^I^ center by a phosphine brace, **C**. Extension of the Si−Si bond compared with free ligand, and a significant donor/acceptor interaction with the Cu^I^ center that is identified computationally (B3PW91/SDD/6‐31G**), signal the formation of a σ‐interaction.

Intermolecular E−E σ‐bond complexes have also been reported. The 3‐coordinate Pt complex, Pt[NHC(Dip)_2_](SiMe_2_Ph)_2_ (NHC=N‐heterocyclic carbene; Dip=2,6‐diisopropylphenyl), **D** has been characterized as being a Pt^0^ σ‐disilane complex (Scheme 8).^[102]^ Computational studies (B3PW91/BS‐II/B3PW91/BS‐I) show that there is significant back donation from the Pt^0^ into the σ* orbitals of the R_3_Si−SiR_3_ ligand that complements donation to the metal from the Si−Si bonding orbital, together resulting in a significant lengthening of the Si−Si bond. Important supporting spectroscopic evidence comes from the ^195^Pt chemical shift that signals a Pt^0^ center rather than Pt^II^.

A platinum complex, Pt(PEt_3_)_2_{η^2^‐(Ph)CC(2,4,6‐Me_3_C_6_H_2_)B(2,4,6‐Me_3_C_6_H_2_)}, **E**,^[103]^ has an unsupported borirene ligand that interacts with the metal center through a B−C single bond, which is significantly lengthened by coordination to the metal center. The bonding is best represented as a Pt^0^ metal center in which donation from a B−C σ bond is supported by Pt to B dative bonding. A base‐stabilized diborane(5) complex of Cu, **F**,^[104]^ features a lengthened B−B single bond that forms a σ‐interaction with the metal (Scheme 8). As for the other E−E σ‐bond complexes, DFT calculations (OLYP/TZ2P) show significant σ‐donation from the B−B bond to the metal center, accompanied by back‐donation to vacant B−B orbitals. While undoubtedly a complex where a B−B single bond interacts with a metal center, this unusual single bond shows significant π‐character. Consequently, it is perhaps not as immediately clear whether this is a true σ‐complex rather than an analogue of Chatt‐Dewar bonding of an alkene (C=C⋅⋅⋅M).

## 3 Examples of the σ‐CAM mechanism

### 3.1 Dynamics and σ‐CAM involving agostic interactions

We begin our survey of examples of the σ‐CAM mechanism with agostic interactions because they were not included in the 2007 review and provide a widespread and productive extension to the concept. (We use the half‐arrow symbol for agostic interactions, Scheme 2.)^[10]^ The central portion of the σ‐CAM mechanism is the dynamic interchange of partners between two σ‐bond ligands. Dynamic rearrangements are typical of agostic complexes, but we need to select examples involving the partner interchange characteristic of the σ‐CAM mechanism.

**Scheme 9:**
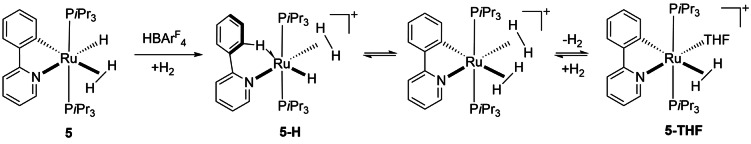
Protonation via an agostic complex and a σ‐CAM mechanism, adapted from ref. [105].

The protonation of the ruthenium complex **5** with [H(OEt_2_)_2_][BAr^F^_4_] in THF (Scheme 9) yields the cyclometalated dihydrogen cation **5‐THF**. If the reaction is performed under dihydrogen, the agostic intermediate **5‐H** may be isolated. This complex loses H_2_ reversibly to form the product. DFT calculations (B3PW91/RECP/6‐31G(*d*,*p*)) indicated that this reaction is triggered by transfer of the agostic aromatic hydrogen to form the bis‐dihydrogen complex via a single TS. Thus the overall reaction conforms perfectly to a σ‐CAM mechanism.^[105]^ Reaction with HBAr^F^_4_ under D_2_ results in exchange of H for D at the ortho positions of the phenyl ring, consistent with this mechanism with the added step of phenyl rotation. This reaction may be considered as a model for the mechanism of the Murai reaction which is catalyzed by Ru(H)_2_(H_2_)_2_(PR_3_)_2_.[^106^, ^107^, ^108^]

**Scheme 10:**
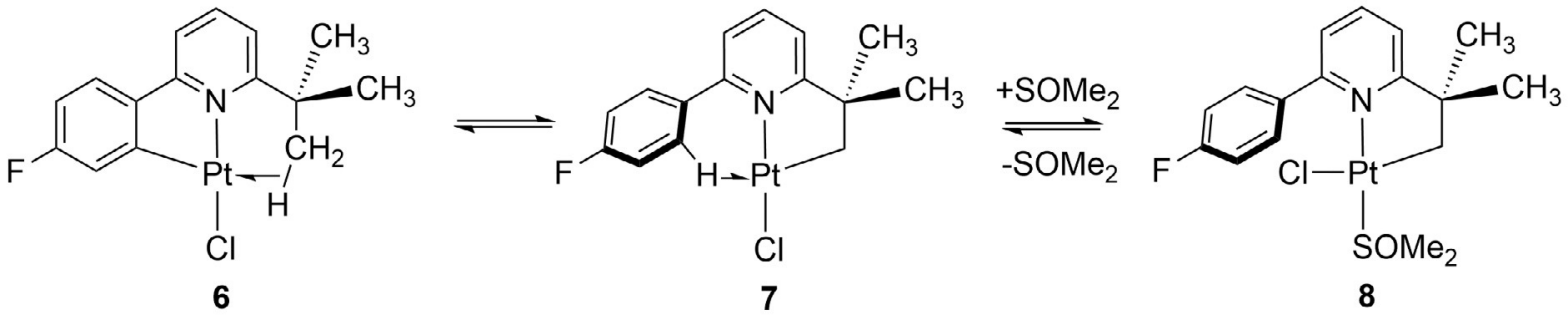
Transformations of agostic complex **6** on reaction with L=DMSO, (adapted from ref. [109]).

The agostic platinum complex **6** is cyclometalated at the aromatic ring but transforms into a complex cyclometalated at the alkyl group **8** on reaction with L = SOMe_2_ (Scheme 10). This reaction is postulated to proceed via the isomer **7** in which the alkyl group is cyclometalated and the aromatic ligand forms the agostic interaction, which is then displaced by SOMe_2_. This isomer **7** lies only 25 kJ mol^−1^ above **6** according to DFT calculations (OPBE, triple ζ, CoSMO).^[109]^ The conversion of **6** to **7** may be an example of an intramolecular σ‐CAM reaction if this is connected by a single TS. A σ‐CAM mechanism was also postulated for the “rollover”^[110]^ reaction of **6** on prolonged reaction with Me_2_SO in which the phenyl pyridine ligand transforms from *C,N* to *C,C* coordination, but no details were provided.

**Scheme 11:**
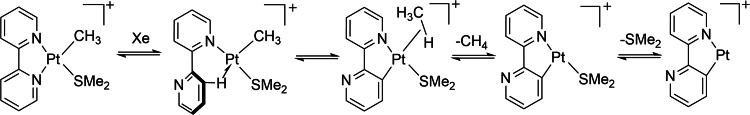
σ‐CAM pathway for gas‐phase rollover of bipyridine employing xenon as the collision gas.^[111b]^

The nature of this rollover process can be understood from the gas‐phase study of the rollover and loss of methane from [Pt(bpy)(SMe_2_)(CH_3_)]^+^ when subject to collision‐induced dissociation conditions.^[111a]^ Scheme 11 shows the σ‐CAM pathway proposed from DFT calculations (B3LYP, TZVP) involving formation of an agostic pyridyl group prior to a σ‐methane complex, but an oxidative addition‐reductive elimination pathway lies close in energy.

A similar σ‐CAM rollover mechanism has been proposed involving an agostic complex and a dihydrogen complex at ruthenium.^[112]^ This mechanism is supported by EXSY NMR experiments showing exchange between the hydride and appropriate CH protons.

**Scheme 12:**
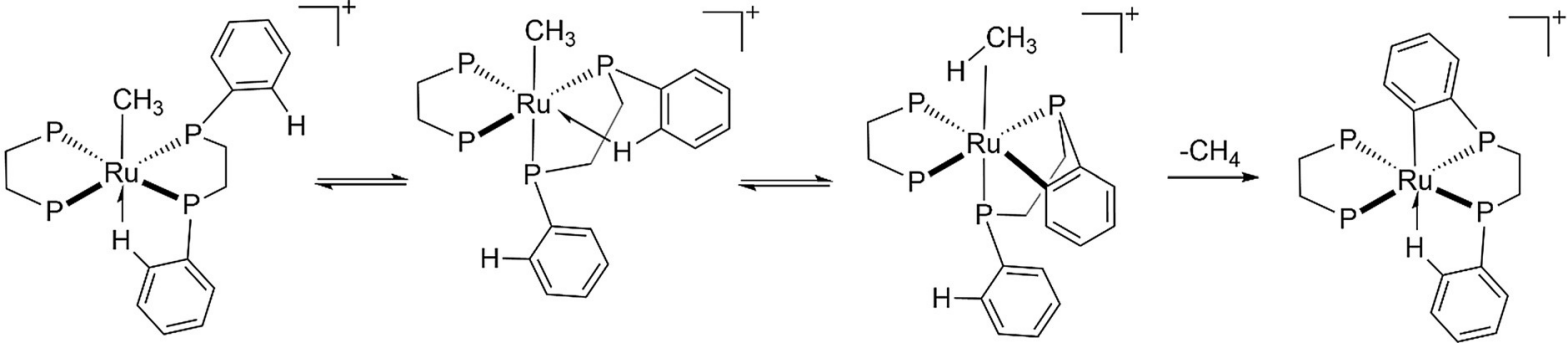
Cyclometalation of dppe at Ru via σ‐CAM (only phenyl groups involved in the transformation are shown), adapted from ref. [113].

The complex [Ru(dppe)_2_Me][OTf] also exhibits an agostic interaction with a phenyl from dppe that positions the agostic interaction trans to the methyl group. This complex undergoes cyclometalation with loss of methane. The reaction proceeds via a σ‐CAM mechanism involving isomerization to place the agostic interaction cis to the methyl group followed by conversion to a methane complex and loss of methane (Scheme 12).^[113]^ This mechanism is supported by the presence of NOE interactions between the methyl group and ortho phenyl protons and by DFT calculations (BSLYP/LANL2DZ or MO6‐L/QZVPPD or PBE/QZVPPD).

A related mechanism has been postulated for the reversible double C−H activation of ethers by (κ^4^‐*N*,*N′,N′′,C*‐Tp^tol′^)Ir(Ph)(N_2_) requiring formation of a σ‐complex with the ether followed by an agostic complex involving coordination of one of the ligand tolyl arms.^[114]^

When Ru(D)_2_(D_2_)_2_(PCyp_3_)_2_ (see section 2.1) is left for a few days in C_6_D_6_ or is pressurized with 3 atm D_2_, H/D exchange occurs, resulting in endo‐selective incorporation of deuterium in the 3‐ and 4‐positions of the cyclopentyl rings, as shown by both NMR spectroscopy and single‐crystal neutron diffraction. DFT calculations (B3PW91/RECP/6‐31G(*d*,*p*)) show that H/D exchange is initiated by isomerization from trans‐ to cis‐phosphines, followed by dihydrogen loss. The C−H activation step at the C3 or C4 position of the ring leads first to an agostic complex, then to a cyclometalated complex with the hydrogen transferred to form a new dihydrogen ligand, thus retaining the Ru^II^ state throughout. Exchange with D_2_ completes the process, which overall is fully consistent with a σ‐CAM mechanism.^[16]^

**Figure 9:**
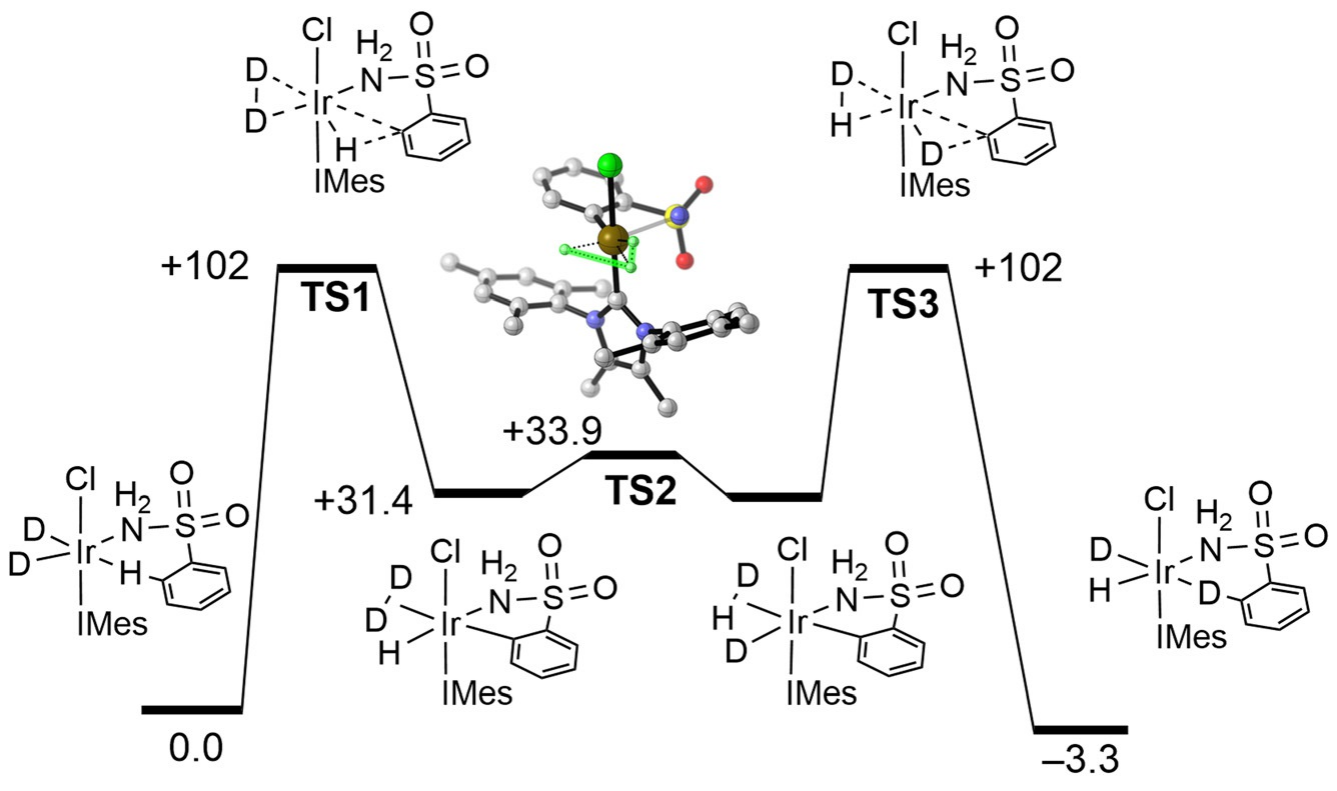
Free energy diagram (kJ mol^−1^) for the selective H/D exchange at constant oxidation state Ir^III^. Inset shows the calculated transition state structure for D_2_/IrH exchange. The entering and leaving ligands are not shown. Adapted from ref. [115] and with thanks to Dr Marc Reid (University of Strathclyde) for providing the inset structure.

The role of agostic interactions in σ‐CAM mechanisms involved in catalytic H/D exchange process has been investigated at Ir^III^ centers.^[115]^ Using the precatalyst Ir(COD)Cl(NHC) (NHC=N‐heterocyclic carbene) and primary sulfonamides as substrates, selective ortho aryl H/D exchange occurs on addition of D_2_. A computational study (M06/6‐31G(d)) shows that this operates through a sequence of steps, at constant Ir^III^ oxidation state, after initial addition of D_2_ to the Ir^I^ center and reduction of the cyclo‐octadiene ligand (not shown). The resulting dideuteride (Figure 9) has an agostic interaction between an *ortho*‐aryl C−H and the Ir^III^ center. This agostic complex connects to an η^2^‐D_2_/aryl hydride intermediate via **TS1** which moves one σ‐interaction (agostic C−H) to another (D_2_) without exchange of partners. A low energy H/D exchange via **TS2** (structure shown in inset) results in a σ‐CAM partner interchange and the formation of η^2^‐H−D and Ir−D. A final exchange of σ‐bonded interactions (**TS3**) leads to selective installation of an *ortho* C−D bond on the substrate.

### 3.2 Alkane C−H σ‐bond complexes in combination with other σ‐bond complexes

**Figure 10:**
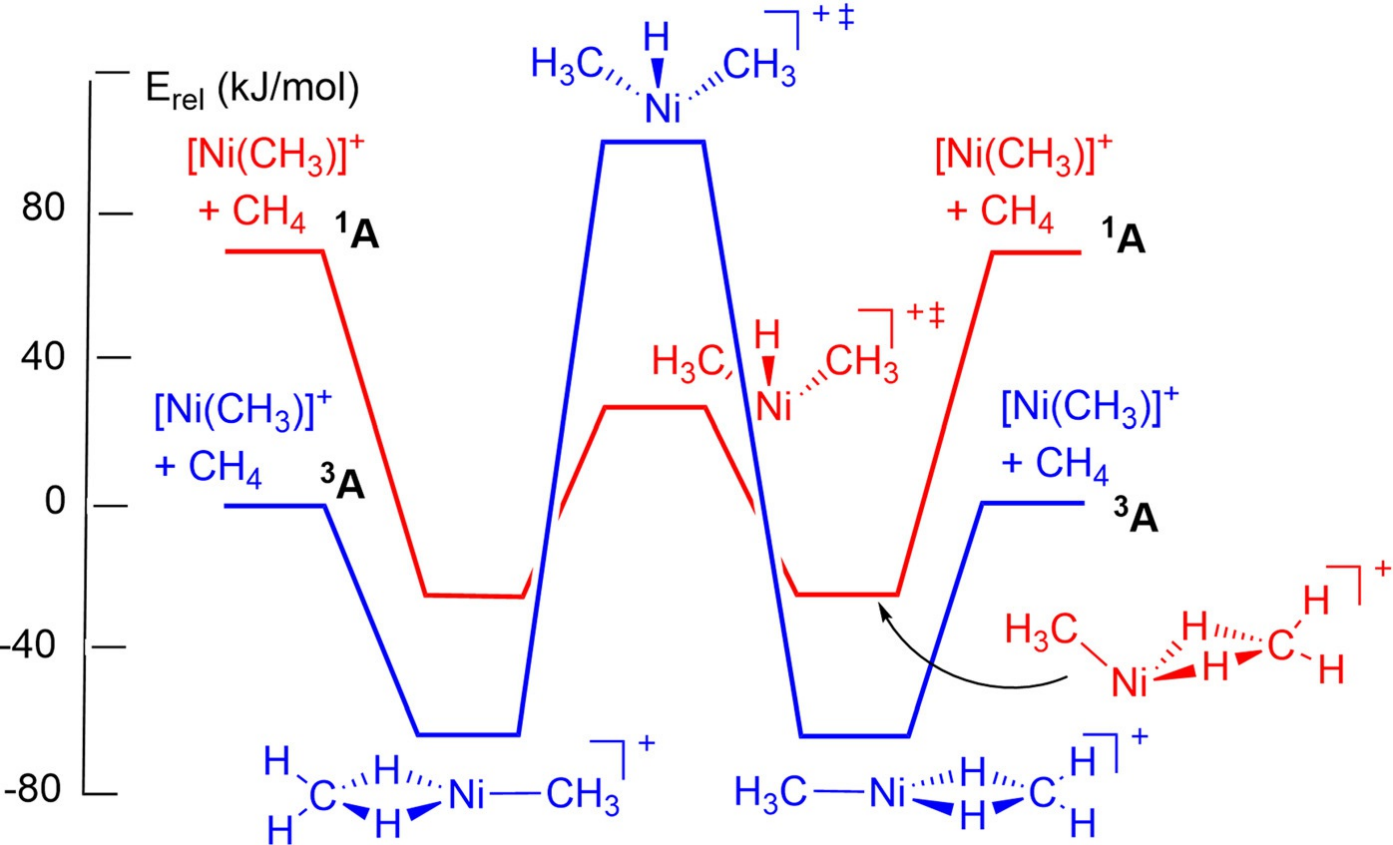
Schematic reaction profiles for the degenerate σ‐CAM reactions of [Ni(CH_3_)]^+^ with CH_4_ in the ^1^A (red) and ^3^A (blue) states of the cation. Adapted from ref. [111].

The degenerate interchange reaction of [M(CH_3_)]^+^ with methane is the simplest possible reaction to test the σ‐CAM mechanism and has been investigated in the gas phase by mass spectrometry and by computation (DFT with B3LYP/TZVP) for metals of groups 8, 9 and 10. The 3*d* metals all undergo interchange via the σ‐CAM mechanism following Scheme 1 a exactly, but the barriers vary with the spin state of [M(CH_3_)]^+^. In the Ni case, the barrier is considerably lower in the singlet manifold than in the triplet; the difference is associated with changes in structure of the [Ni(CH_3_)(CH_4_)]^+^ intermediate and the TS (which have oxidative cleavage character, Figure 10). Among the 4*d* metals, Ru and Rh clearly prefer oxidative addition‐reductive elimination, while for Pd the preference is marginal.^[111b]^

**Scheme 13:**
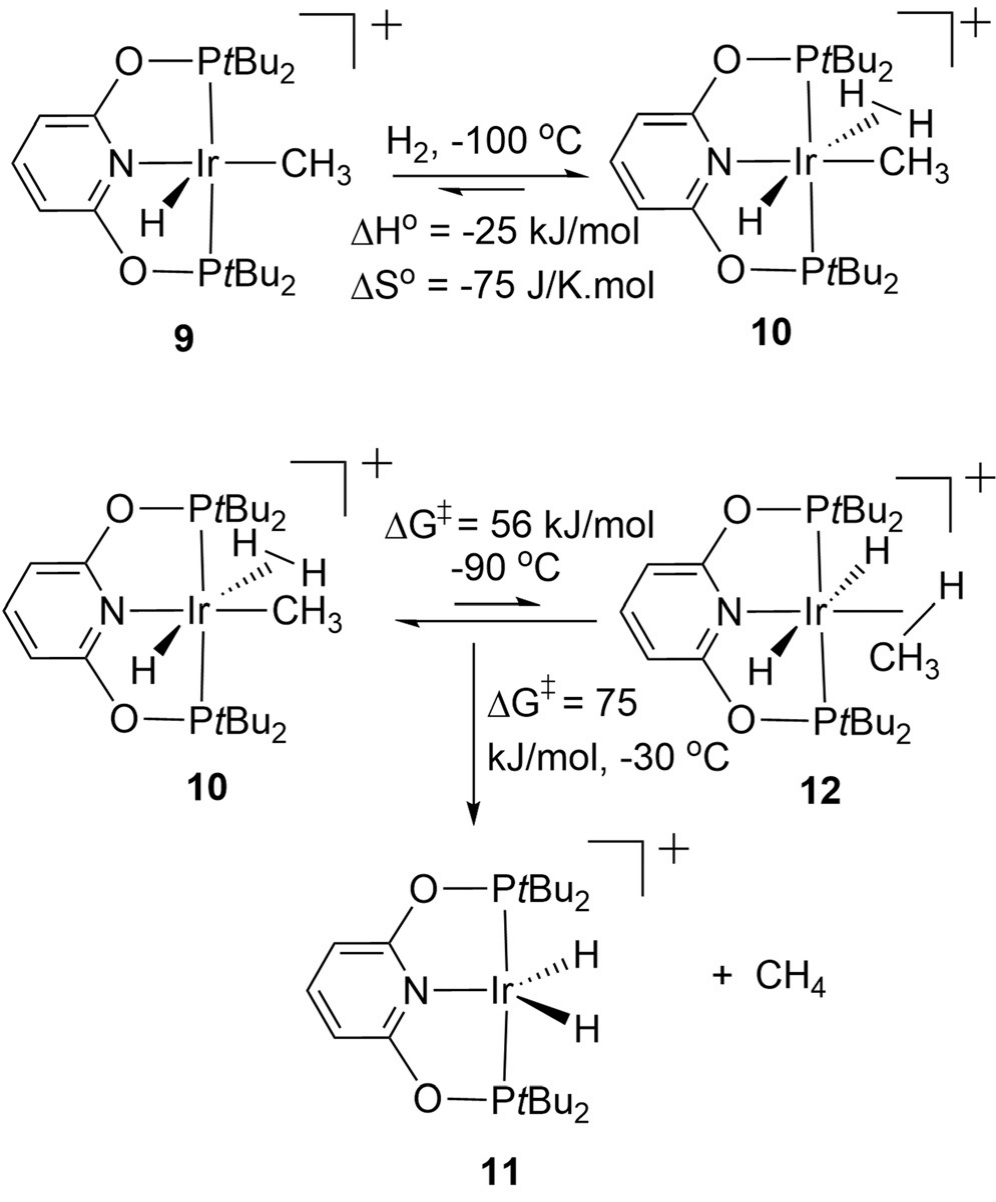
Hydrogenolysis of an iridium methyl hydride complex with experimental energetics and activation barriers.^[39]^

An exceptionally clear‐cut example with both experimental and computational evidence for a σ‐CAM process is provided by the hydrogenolysis of an iridium methyl pincer complex.^[39]^ Dihydrogen adds to [Ir(PONOP)(CH_3_)H]^+^ **9** (PONOP=2,6‐bis(di‐*t*‐butylphosphinito)pyridine) at its vacant site at −100° C to generate a dihydrogen complex **10** which is in equilibrium with the precursor. On warming, these species are replaced by the dihydride complex **11** with evolution of methane (Scheme 13). However, if the reaction is performed with D_2_, exchange is observed at −90° C into the coordinated methyl group and the terminal hydride at equal rates, providing decisive evidence for an equilibrium between **10** and the isomeric methane complex **12**. It was possible to estimate by experiment both the barrier to exchange between **10** and **12** and the barrier to loss of methane and formation of **11**. This process was modelled by DFT (PBE0/6‐311G**) successfully as direct conversion of **10** to **12** with no intermediate (i.e. a single TS) with a barrier within 1.3 kJ mol^−1^ of the observed barrier, entirely consistent with a σ‐CAM mechanism.^[39]^

**Scheme 14:**
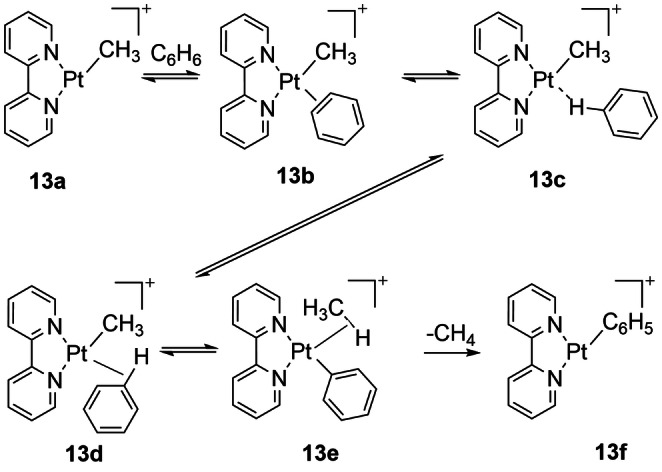
Dissociation of methane from Pt(bpy)(CH_3_)]^+^ by CID in gas phase. Note formation of σ‐complex with benzene.^[116]^

The C−H activation of benzene by [Pt(CH_3_)(2,2′‐bpy)]^+^ (**13 a**) has been studied mass spectrometrically in the gas phase using collision‐induced dissociation (CID) methods including use of deuterated isotopologues. It undergoes dissociation of methane at low collision energies without cyclometalation. DFT calculations with the mPW1k functional favor the σ‐CAM pathway and explain the H/D exchange behavior (Scheme 14). This example illustrates the extension of the σ‐CAM concept to include the σ‐coordination of a C−H bond of an arene, here benzene itself. The benzene is initially coordinated in a π fashion (**13 b**) and then moves to σ‐coordination (**13 c/13 d**, barrier 71 kJ mol^−1^) before isomerizing to the η^2^‐*C,H*‐methane complex (**13 e**) and finally losing methane (barrier 60 kJ mol^−1^).^[116]^ However, use of another functional (M05‐2X) makes the σ‐CAM barriers essentially the same as the oxidative‐addition/reductive elimination barrier.

Other examples involving σ‐alkane complexes include [Ru(dppe)_2_Me][OTf] requiring agostic and η^2^‐methane complexes (see Scheme 12)^[113]^ and cobalt hydrosilation requiring dihydrogen, η^2^‐silane and η^2^‐alkane complexes (see Scheme 17).^[117]^

### 3.3 Silane and germane σ‐bond complexes in combination with other σ‐bond complexes

**Scheme 15:**
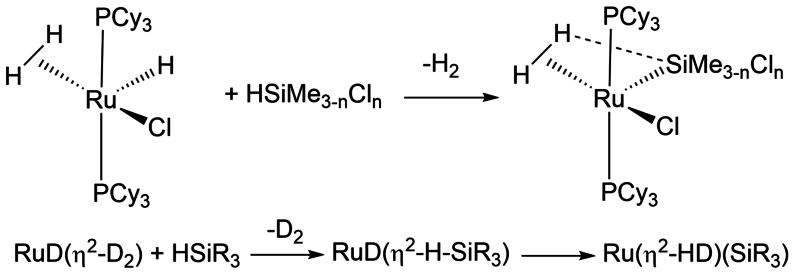
Formation of ruthenium silyl complex from chlorosilanes and σ‐CAM sequence for D_2_ isotopologue.^[118]^

One of the simplest reactions of silanes involving the σ‐CAM mechanism is shown in Scheme 15.^[118]^ Here, the 16‐electron RuH(H_2_) species reacts with hydrosilane (HSiMe_2_Cl, HSiMeCl_2_ or HSiCl_3_) to form a Ru(H_2_)(SiMe_3−*n*_Cl_*n*_) product. The crystal structure and DFT calculations (B3PW91) for the Ru(H_2_)(SiMeCl_2_) product provide strong evidence for a close approach of one hydrogen to silicon (a SISHA interaction). The calculations show that the η^2^‐silane isomer RuH(η^2^‐HSiMe_3−*n*_Cl_*n*_) lies very close in energy to the dihydrogen complex and may even be preferred in one rotamer of the SiMe_2_Cl complex. When the initial reaction is performed with the RuD(D_2_) complex, the product is Ru(HD)(SiMe_3−*n*_Cl_*n*_). All these observations are consistent with the σ‐CAM sequence shown in Scheme 15.

**Scheme 16:**
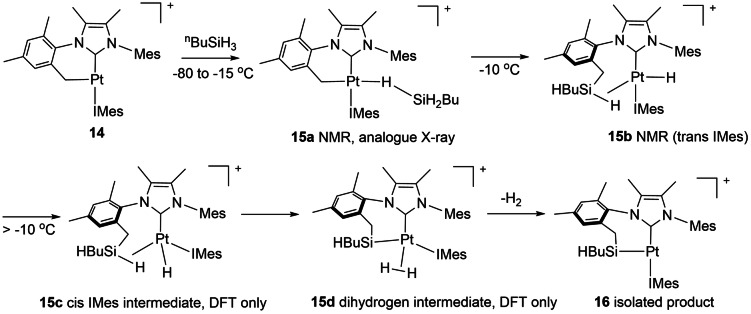
SiHBu insertion into a Pt−C bond with σ‐CAM step converting **15 c** to **15 d**.^[119]^

In a well characterized sequence, the T‐shaped cyclometalated platinum cation **14** reacts with primary silanes to form a C−Si−Pt linkage **16** via two intermediates, characterized at low temperature (Scheme 16).^[119]^ The first intermediate, **15 a** contains an η^1^‐SiH_3_R group; it exhibits an SiH proton with large couplings to ^29^Si and ^195^Pt and has been characterized crystallographically for R=Ph and by NMR spectroscopy for R=^n^Bu. In the second intermediate, **15 b**, the C−SiH_2_R bond has formed and there is an η^2^‐SiH link to Pt (also observed for R=^n^Bu). In the next intermediate, **15 c** characterized by DFT (M06/SDD/6‐31G(*d*,*p*)/SMD), a trans‐cis isomerization has occurred before a σ‐CAM step yields a dihydrogen complex,**15 d**. Finally, H_2_ is lost to form the product. According to the calculations, the conversion of **15 a** to **15 b** involves an oxidative addition to form a Pt^IV^ intermediate and should not be termed a σ‐CAM mechanism, although this intermediate lies in a very shallow minimum. The conversion **15 c** to **15 d** is a σ‐CAM reaction according to the calculations.

**Scheme 17:**
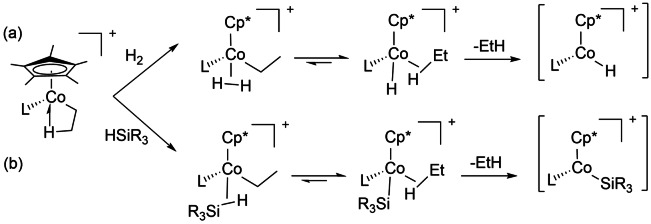
Cobalt σ‐complexes and their σ‐CAM interconversion in initiation of (a) hydrogenation, (b) hydrosilation.^[117]^

The cobalt agostic ethyl cations, [Cp*Co(CH_2_CH_2_‐μ‐H)(L)]^+^ (L=PMe_3_, P(OMe)_3_) are isolable as [BAr^F^_4_]^−^ salts and undergo reaction with dihydrogen to form well‐characterized η^2^‐H_2_ complexes and with silanes to form η^2^‐silane complexes. The agostic salts also catalyze alkene hydrogenation and hydrosilation. The combination of these three groups of σ‐complexes led the authors to propose that the catalytic hydrogenation is enabled by a σ‐CAM mechanism in which the agostic cation is converted first to a dihydrogen cation and then an η^2^‐ethane cation, before ethane is lost to create a coordinatively unsaturated hydride cation (Scheme 17 a). Similarly, hydrosilation is initiated by formation of an η^2^‐silane complex followed by an ethane complex generating the unsaturated silyl cation and ethane (Scheme 17 b). In catalytic polymerization of alkenes, related mechanisms may also feature in chain transfer or termination by reaction with dihydrogen or silane.^[117]^

A germane σ‐complex was first characterized crystallographically in 2003,^[120]^ and further examples are reviewed in ref. [121]. In comparison to silicon, there is a shift from σ‐coordination toward oxidative addition. A σ‐CAM mechanism has been postulated in the reaction of Ru(H)_2_(H_2_)_2_(PCy_3_)_2_ with GeH_2_Ph_2_ to form a germylene complex.^[122]^

### 3.4 B−H σ‐bond complexes in combination with other σ‐bond complexes

We have already shown that 3‐ and 4‐coordinate boranes have a rich coordination chemistry associated with σ‐complex formation. They also undergo a wide variety of bond activation processes in which σ‐CAM mechanisms operate, and these are discussed in this section. We highlight three examples where σ‐CAM mechanisms clearly operate, that cover B−H activation. Diboranes, such as R_2_B−BR_2_ also undergo bond activation processes where σ‐CAM mechanisms may operate.^[123]^

**Scheme 18:**
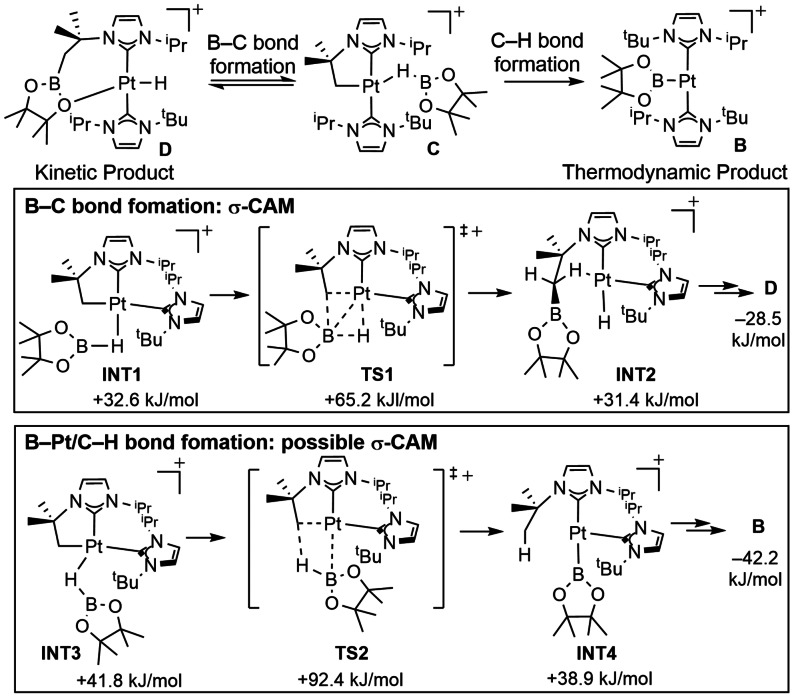
Kinetic and thermodynamic products in the reaction of [Pt(I^t^Bu^i^Pr′)(I^t^Bu^i^Pr)][BAr^F^_4_] with HBpin leading to B−C bond formation.^[124]^

A coordinatively unsaturated cationic Pt^II^ complex related to that in Scheme 16, provides a very well‐defined system where σ‐CAM mechanisms operate in B−C and C−H bond formation processes.^[124]^ An initial complex [Pt(I^*t*^Bu^*i*^Pr′)(I^*t*^Bu^*i*^Pr)][BAr^F^_4_] **A** (I^*t*^Bu^*i*^Pr=1‐tert‐butyl‐3‐isopropylimidazol‐2‐ylidene, I^*t*^Bu^i^Pr′=its ^*t*^Bu cyclometalated form) reacts with HBpin (pinacolborane) to ultimately form the Pt‐boryl complex [Pt(I^*t*^Bu^*i*^Pr)_2_(Bpin)][BAr^F^_4_], **B** (Scheme 18). Low temperature NMR spectroscopy studies show the initial formation of the σ‐bond complex [Pt(I^*t*^Bu^i^Pr′)(I^*t*^Bu^*i*^Pr)(η^1^‐HBpin)][BAr^F^_4_], **C**, for which a close analogue has been crystallographically characterized. At low temperature, **C** then undergoes a B−C coupling process to form the hydride complex [PtH(I^*t*^Bu^*i*^PrBpin)(I^*t*^Bu^*i*^Pr)][BAr^F^_4_] **D**. This B−C bond formation is reversible, and on warming the thermodynamic product **B** prevails, which results instead from C−H bond formation. A detailed computational study (M06/BS3) supports this experimentally determined reaction landscape, as well as signposting possible σ‐CAM processes. For the (reversible) B−C bond forming step, a σ‐bound HBpin complex, **INT1** (an isomer of **C**), moves through a transition state, **TS1**, which forms a new Pt^II^ intermediate, **INT2**, with an agostic Pt⋅⋅⋅H‐C interaction: a clear σ‐CAM. This intermediate ultimately (reversibly) isomerizes to give the kinetic product, **D**. The thermodynamic product, **B**, arises from another isomer of **C**, the σ‐bound HBpin complex **INT3**, but now a new C−H bond is formed at a Pt^II^‐boryl complex, **INT4**, via **TS2** before isomerizing to form **B. INT3** to **INT4** is also a σ‐CAM sequence if **INT4** has an agostic C−H interaction, although the structure is not discussed in detail by the authors.

**Scheme 19:**
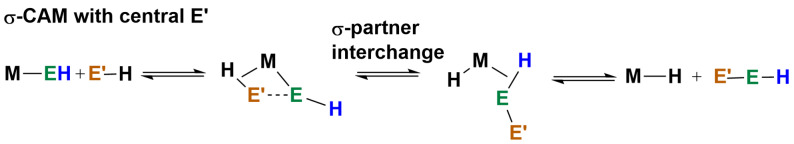
Variant of σ‐CAM mechanism with E′ occupying the central position.

The conversion of **INT1** to **INT2** represents a rare example in which the central position of the partner interchange typical of the σ‐CAM mechanism is occupied by boron and not hydrogen. Nevertheless, the **INT2** contains an agostic link to a C−H bond and not an η^2^‐B−C bond. This sequence is characteristic of such σ‐CAM mechanisms with E or E′ occupying the central position as illustrated in Scheme 19.

**Scheme 20:**
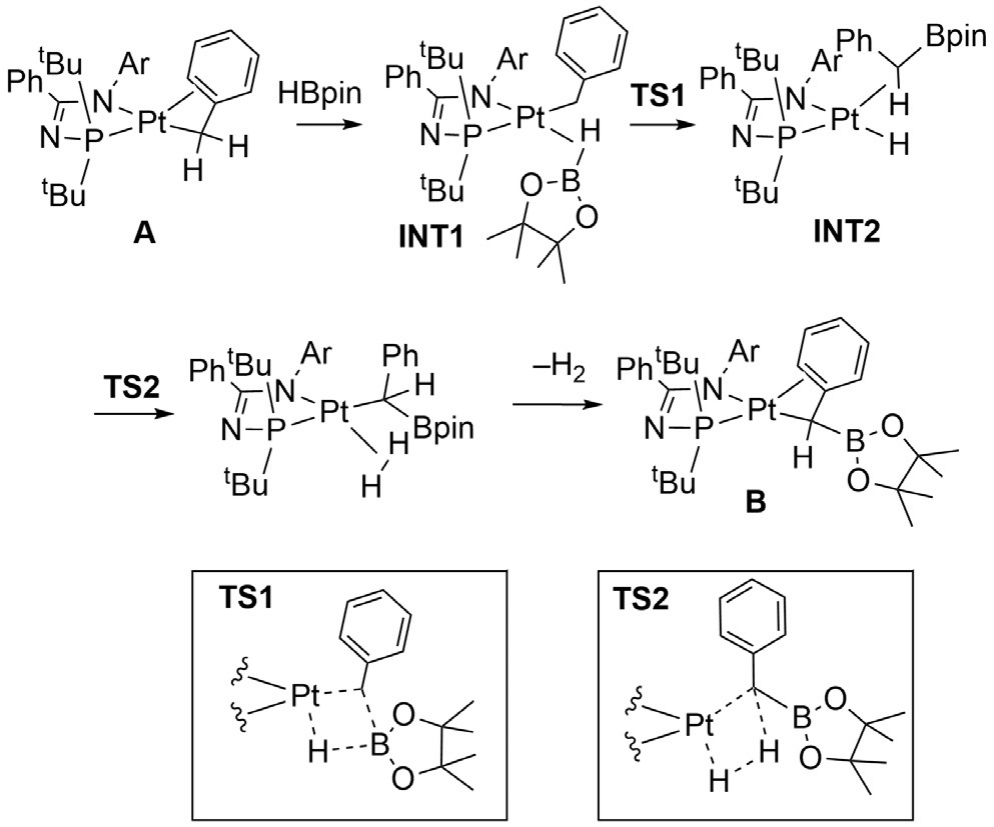
Dehydrogenative benzylic borylation via two sequential σ‐CAM steps.^[125]^

Similar B−H activation/B−C bond‐forming reactivity has been reported at Pt^II^ centers, this time by sequential σ‐CAM steps in a dehydrogenative benzylic borylation reaction (Scheme 20).^[125]^ The 16‐electron Pt^II^ complex **A**, Pt(κ^2^‐P,N)(η^3^‐benzyl) (PN=N‐phosphinoamidinate) reacts with HBpin to cleanly afford Pt(κ^2^‐P,N)(η^3^‐PhCH(Bpin)), **B** with loss of H_2_. Computational studies on the reaction pathway (M06/def2‐TZVP//M06/LANL2DZ[6‐31G**]) indicate the initial formation of a σ‐bond HBpin complex, **INT1**, that undergoes a σ‐CAM process (**TS1**) with an η^1^‐benzyl group to form the Pt^II^ σ‐boratoalkane complex **INT2**. A further σ‐CAM process (**TS2**) results in the formation of a weakly coordinated Pt(H_2_) complex, that loses H_2_ to give **B**. Scheme 20 shows this pathway and the two calculated transition states.

**Figure 11:**
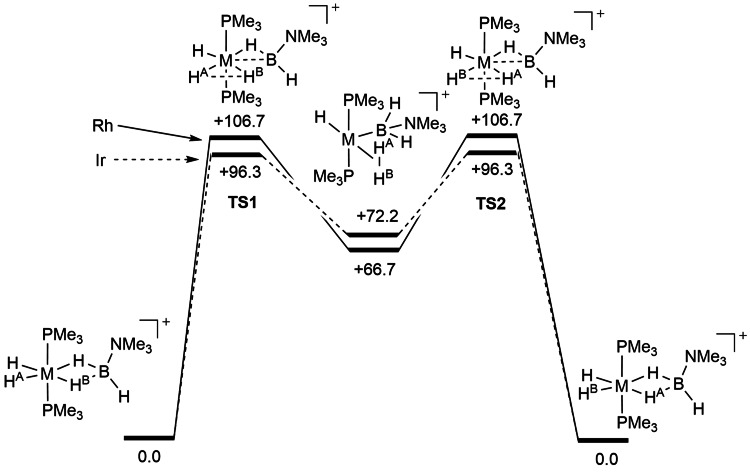
Computed free energy profile for the degenerate hydrogen exchange between M−H and κ^1^,κ^1^‐MH_2_B in the complexes [M(PCy_3_)_2_(H)_2_(η^2^η^2^‐D_3_BNMe_3_)][BAr^F^_4_] (M=Rh or Ir). Dotted lines connect the M=Ir intermediates and transition states, solid M=Rh. Adapted from ref. [84].

The well‐defined amine–borane σ‐bond complexes, [M(PCy_3_)_2_(H)_2_(η^2^,η^2^‐D_3_B⋅NMe_3_)][BAr^F^_4_] (M=Rh or Ir) undergo intramolecular exchange between the M−H and M−D−B bonds, allowing for the effect of the metal to be probed with regard to mechanism and relative barriers. Experimentally, exchange is shown to proceed much faster for M=Ir. Computational studies (BP86/6‐31G**) on the all‐hydrogen system show that interchange operates via a σ‐CAM mechanism (Figure 11).^[84]^ **TS1** connects the starting amine‐borane σ‐bond complex with a dihydrogen base‐stabilized boryl intermediate. An equivalent transition state then connects to the product, in which M−*H* and M−*H*−B have exchanged. While the metal center remains at a constant oxidation state in the intermediates, the TS has some M^V^ character (a relatively short M−H contact for the transferring hydrogen). Consequently, the barrier to exchange is calculated to be lower for 5*d* iridium than 4*d* rhodium, as observed experimentally.

### 3.5 σ‐bound ligands at nanoparticles

The definitive characterization of chemisorbed molecules and atoms on surfaces is significantly more challenging than for molecular complexes. Despite this, there is growing direct evidence for σ‐bond complex formation at metal and nanoparticle surfaces^[126]^ as we have discussed in section 2.2. Here we briefly discuss whether such species can also be implicated in σ‐CAM processes.

**Figure 12:**
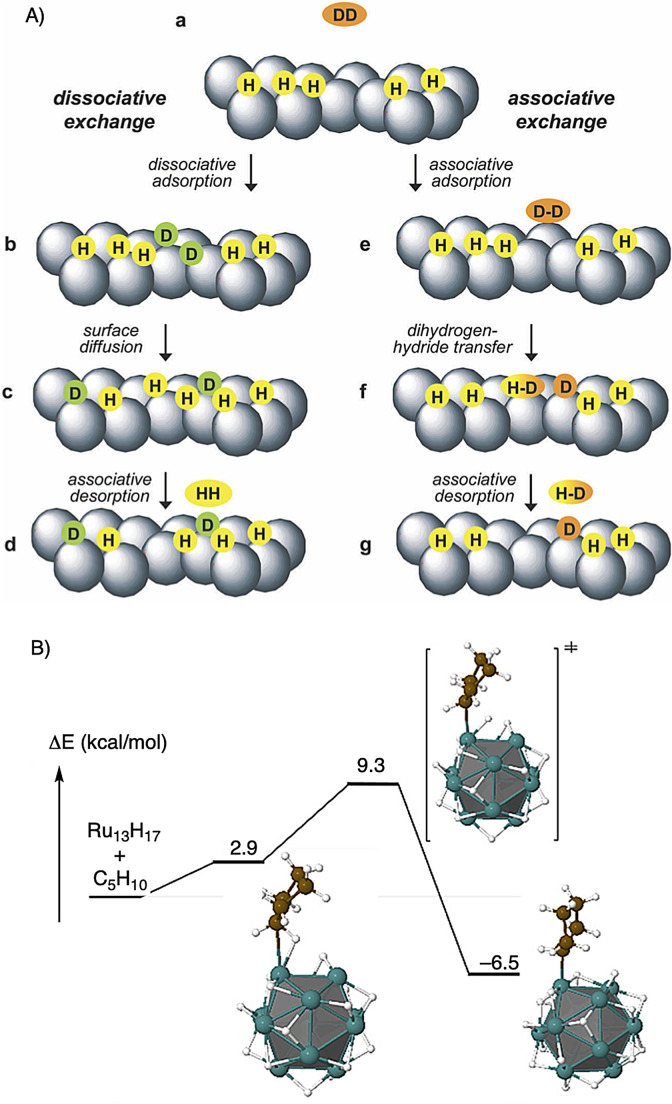
σ‐complexes on Ru nanoparticle surfaces in exchange processes. (A) “Dissociative” and the favored “Associative” mechanisms for H/D exchange between H_2_ and D_2_. Reproduced with permission from ref. [127]. (B) Calculated C−H activation pathway for cyclopentane activation at Ru_13_H_17_. Adapted, with permission, from ref. [52].

A detailed kinetic and spectroscopic analysis has led to the postulate that H/D exchange between H_2_ and D_2_ to form HD occurs on the surface of Ru metal nanoparticles via a, so‐called, associative exchange (Figure 12 A).^[127]^ This invokes a σ‐bond interaction of D_2_ with a metal surface already covered in metal hydride groups. Dihydrogen/hydride transfer—presumably via 3‐center D⋅⋅⋅D⋅⋅⋅H transition state—then leads to surface‐bound HD, followed by desorption into the gas phase. An alternative mechanism, based upon initial D−D bond scission to form surface‐bound deuterides coupled with exchange mediated by fast surface diffusion, was discounted on the basis of careful kinetics measurements of the head‐space of the HD that is formed, using gas‐phase NMR spectroscopy.^[127]^ While this “associative” mechanism captures the essential elements of a σ‐CAM process (σ‐bond intermediates bookending a metathesis process) details of the transition state that connects them are still to be determined.

The same groups have reported a closely related H/D exchange of the C−H bonds of cyclopentane on the surface of Ru nanoparticles with D_2_. Using DFT calculations (PBE) on a model Ru_13_H_17_ system (Figure 12 B), this exchange is proposed to occur via σ‐alkane complex formation, followed by C−H activation that forms a Ru−C bond reversibly.^[52]^ While no details of the bonding mode of the released hydrogen were disclosed, it is tempting to speculate that a surface‐bound η^2^‐H_2_ species is possible, which would then characterize the exchange process as a σ‐CAM process. For both surface processes described here, it will be interesting to see if future computational work can provide more detail on σ‐bound intermediates and the linking transition states.

## 4 Assessment of extensions of the original σ‐CAM concept

In this section, we summarize and assess the extensions to the original σ‐CAM concept that we have highlighted in the preceding sections providing cross‐references to the appropriate schemes and figures.

### 4.1 Additional element‐hydrogen and element–element bonds

In our original review, we provided evidence for the operation of σ‐CAM for H−H, C−H, Si−H and B−H bonds. The current review provides some evidence for η^2^‐Ge−H bonds in σ‐CAM processes (sections 3.3). The involvement of B−H bonds is now seen to include the η^2^,η^2^‐*B,H*‐BH_3_L (L=amine) coordination as well as the η^2^‐*B,H*‐BHR_2_ (Figure 11). Given the clear evidence of σ‐coordination of Zn−H, Al−H and Ga−H (section 2.5), we can anticipate further examples of σ‐CAM involving groups 12 and 13. Similarly, the isolation of σ‐E−E′ complexes (E−E′=B−C, C−C, Si−C, B−B; section 2.6) provides a basis for the potential involvement of E−E bonds in σ‐CAM processes.

### 4.2 Agostic complexes

In section 3.1, we provided several examples illustrating σ‐CAM reactions resulting in conversion of an agostic complex to a complex with an η^2^‐E−H bond (Schemes 9–12, Figure 9). Further examples, such as that shown in Scheme 16 demonstrate parallel events for SiH‐agostic complexes. Our 2007 review only gave examples of intermolecular metathesis, but the examples of agostic interaction illustrate intramolecular sources for the incoming or outgoing ligand. Similarly, the order of events shown in Scheme 1 a may be varied according to the electron configurations and structures of the participating molecules. For example, Scheme 12 conforms to the standard order: new ligand enters–σ‐partner interchange–ligand leaves. That is not the case for Scheme 10 where we see σ‐partner interchange–ligand leaves–new ligand enters.

### 4.3 Extension to coordination of sp^2^‐carbon

The examples of σ‐CAM mechanisms involving C−H bonds were originally confined to *sp* ^3^‐carbon with alkyl groups or alkanes. The demonstration of comparable mechanisms involving agostic phenyl groups demonstrates that the principle can be extended to *sp* ^2^ ‐carbon in the form of (η^1^‐*H*,*C*‐aryl) (Schemes 9–13 in section 3.1). There are numerous examples of isolable M(η^2^‐*C,C*‐arenes)[^128^, ^129^] and some of early metal M(κ^1^‐*F,C*‐arene) coordination.^[130]^ However, intermolecular coordination of arenes via M(η^1^‐*H,C*‐arene) or M(η^2^‐*H,C*‐arene) linkages is frequently postulated via DFT calculations, for example between TpW(PMe_3_)(NO)(η^2^‐C_6_H_6_) and TpW(PMe_3_)(NO)(Ph)(H) complexes.^[129a]^ An intermolecular η^2^‐Rh⋅⋅⋅H−C(aryl) σ‐interaction has been observed in the solid state.^[129b]^ Thus, it seems reasonable to postulate a σ‐CAM process involving M(η^1^‐*H,C*‐arene) or M(η^2^‐*H,C*‐arene).

### 4.4.‐Extension to surfaces

In section 3.5, we summarized evidence for reactions at nanoparticle surfaces involving σ‐complexation (Figure 12). Given the encouraging clues above, we suggest that σ‐CAM processes are possible in E−H bond activation that occurs at surfaces.

## 5 Critical overview

The most compelling examples of σ‐CAM highlighted in this review include a combination of extensive experimental and computational evidence. Good experimental evidence for the σ‐complexes and their interconversion by σ‐CAM processes constrains the computational studies to realistic energy landscapes. Once suitably calibrated, computation identifies transition states and associated barriers that link the component intermediates of the reaction pathway. Such examples that include the characteristics of interchange of σ‐partners at constant oxidation state through a single TS are illustrated in Schemes 11, 13, 16–18 and Figures 11, 12.

Delineation of the σ‐CAM pathway benefits from the ability to isolate (or just detect, either directly through experiment or indirectly through computation) the σ‐complex intermediates and their interconversion for late transition metals, especially with *d* ^6^ or *d* ^8^ configurations. The σ‐CAM mechanism shares with oxidative cleavage/reductive coupling and 1,2‐addition (Scheme 1) the focus on reaction intermediates, rather than transition states. In contrast, the σ‐bond metathesis mechanism, so characteristic of *d* ^0^ configurations, does not require reaction intermediates. Although sigma complexes may be spotted as shallow minima in DFT calculations at *d* ^0^ (summarized by ref. [131]), they are rarely, if ever, detected experimentally.

σ‐Bond metathesis and 1,2‐addition are not confined to *d* ^0^ systems. The role of polar metal‐heteroatom bonds with a lone pair in 1,2‐addition has been analyzed for *d* ^0^, *d* ^6^ and *d* ^8^ configurations and compared to σ‐bond metathesis or oxidative addition to metal‐centers without a suitable heteroatom. The comparisons lead to the headline conclusion that facile metal‐mediated 1,2‐C−H addition requires a strong σ‐accepting orbital on the metal and a polar M−X bond that has substantial electron density. Thus 1,2‐additon should be considered as an alternative to σ‐CAM when these features are present.^[132]^

Another question arises when the possibility of a σ‐CAM mechanism is discussed: when will oxidative cleavage/reductive coupling be preferred to σ‐CAM? One approach to answering this question is to compare the energies of σ‐complexes to their oxidative cleavage analogues. Two examples demonstrate the subtlety of this issue. The σ‐methane complex of [Rh(PONOP)]^+^ lies below the corresponding rhodium methyl hydride complex. For iridium, the reverse is true: the iridium σ‐methane complex lies at higher energy as revealed by EXSY NMR experiments.[^38^, ^133^] Alkane (ethane and methane) and alkyl hydride complexes of CpRe(CO)_2_ and Cp*Re(CO)_2_ have been detected directly by TRIR spectroscopy in supercritical methane and ethane.^[134]^ In this example, the σ‐complex and the oxidative‐cleavage product are at equilibrium. The position of equilibrium shifts toward the alkane complex with the Cp analogue and with use of ethane instead of methane. Thus small changes may induce a change in the ground state structure and/or barriers to kinetically accessible bond activation/formation partners. In turn, these changes will affect the mechanistic path taken.

The capability of investigating the structure of transition states by computational methods has led to alternative definitions of mechanism based on transition‐state geometries that are unobservable using experimental methods. However, these definitions do not restrict the identity of the intermediates on either side of the TS. Such mechanistic nuances that are relevant to the current review include metal‐assisted σ‐bond metathesis (MAσBM),[^135^, ^136^] oxidatively‐added transition state (OATS),^[137]^ oxidative hydrogen migrations (OHM)[^138^, ^139^] and ligand‐to‐ligand hydrogen transfer (LLHT).^[140]^ There is a very close relationship of several of these mechanisms to one another that may be differentiated by Bader's Atoms in Molecules (AIM) methods.^[141]^ A further study covering a wide range of electron configurations shows that there is a continuum of charge‐transfer stabilization during C−H activation of methane from electrophilic through to nucleophilic. Moreover, this applies regardless of whether the mechanism is oxidative cleavage or σ‐bond metathesis.^[142]^ Most importantly, a variety of transition states are compatible with the σ‐CAM mechanism, since σ‐CAM defines the intermediates and not the TS. The corollary of this principle is that a particular bond activation process can correspond to both σ‐CAM (defined by σ‐complex intermediates) and one defined by the TS.

To illustrate this concept, the MAσBM mechanism is a version of the σ‐CAM mechanism in which the TS maintains some bonding between the hydrogen undergoing transfer and the metal, as well as the donating and accepting atoms.[^135^, ^136^] The OATS mechanism can also be considered as a version of the σ‐CAM mechanism in which the TS corresponds to oxidative cleavage.^[137]^

Unlike the preceding mechanisms, OHM and LLHT were recognized in systems in which a π‐bonded ligand with *sp* ^2^ or *sp* C−H bonds undergoes metal‐mediated hydrogen transfer. OHM is a C−H activation mechanism identified in catalytic hydroarylation that links an *sp* ^2^ C−H bond of a coordinating benzene or alkene to an alkyl ligand through a TS in which the H is bonded to the metal, but not to either donor or acceptor carbon.[^138^, ^139^]

**Figure 13:**
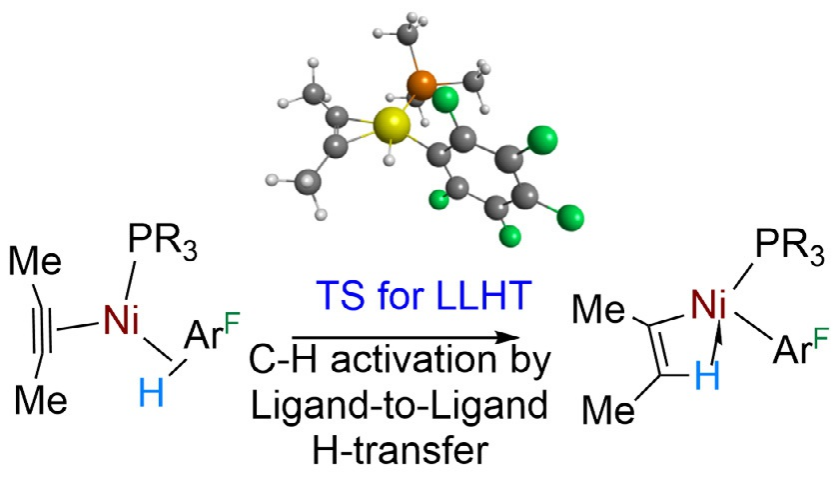
Ligand‐to‐ligand hydrogen transfer (LLHT) mechanism for hydroarylation of an alkyne at nickel showing the TS for LLHT (B3PW91/RECP/6‐31G(*d,p*)). Adapted with permission from ref. [140].

A further related mechanism is ligand‐to‐ligand hydrogen transfer (LLHT). The characteristics of the LLHT transition state are very weak M⋅⋅⋅H interaction and a C_donor_⋅⋅⋅H⋅⋅⋅C_acceptor_ angle close to 180°. The transfer can therefore be viewed as a proton transfer between the two ligands at the transition state.^[140]^ In combination with an entering and leaving ligand, LLHT becomes a metathesis process. This mechanism was first classified for the hydrofluoroarylation of an alkyne. A fluoroarene coordinates to a nickel(II) alkyne complex to form a σ‐C−H complex which transfers hydrogen directly to the alkyne without an intervening hydride, generating an agostic vinyl group (Figure 13). In other examples, hydroarylation of nickel(0) alkene generates an agostic alkyl nickel(II) product by LLHT.[^143^, ^144^] Although the central process of LLHT can be viewed as a proton exchange, there may be a change in oxidation state. This contradiction is apparent rather than real since the proton‐accepting alkyne or alkene ligand lies close to the metallacycle limit in its bonding, and so it can be considered as a Ni^II^ to Ni^II^ process. In those cases where a σ‐complex lies either side of the LLHT transition state, we may also describe the overall metathesis mechanism as a σ‐CAM process.

An earlier example of LLHT may be the protonation of Pt(NN)(C_6_H_5_)_2_ generating [Pt(NN)(C_6_H_5_)(η^2^‐C_6_H_6_)]^+^ (NN=ArN−CMe−CMe=NAr, Ar=2,6‐Me_2_C_6_H_3_) which undergoes hydrogen exchange between C_6_H_5_ and C_6_H_6_ groups according to EXSY experiments, followed by associative displacement of benzene.[^145^, ^146^]

Comparisons between these different mechanisms have also been explored by following the displacements of the centroids of localized molecular orbitals (CLMO) that can help to visualize the mechanisms in the traditional terms of electron pair movements, more commonly known as curly arrows.^[147]^ Examples include the σ‐CAM mechanism of Scheme 16 and a contrasting example of OHM.

The central interchange process in σ‐CAM involves stretching of the coordinated E−H bond and compression of the distance between E′ and the transferring hydrogen atom. This can often be observed as a dynamic interchange process by NMR spectroscopy. Important developments in computation now allow calculation of reactions dynamics, not just for the interchange but for the complete metathesis. Reaction trajectories for several metathesis mechanisms including σ‐CAM, oxidative addition/reductive elimination and σ‐bond metathesis of the types shown in Scheme 1 have been calculated using structures calculated by DFT combined with quasi‐classical dynamics.[^131^, ^148^, ^149^] In these calculations, the trajectory is calculated starting from the TS moving either toward product or in the reverse direction toward the precursor. The authors recognize the limitation that their calculations do not include the effect of solvent. Their conclusion is that the minima defining σ‐complex intermediates may be skipped if the minima are shallow (as for instance in *d* ^0^ or *d* ^6^ σ‐complexes) and there is sufficient energy in the system. This principle is analogous to a person skiing downhill who passes through a shallow dip and continues downhill without stopping. Alternative methods for molecular dynamics including explicit solvent have been explored for transfer hydrogenation.^[150]^ New methodologies involving dynamics are set to enlighten us in the future about the relationship between the different mechanisms of Scheme 1. At present, we infer that mechanistic deductions are more reliable when made from a combination of experimental detection of reaction intermediates and calculation of potential energy surfaces.

## 6 Conclusions

In conclusion, the σ‐CAM concept represents an instructive approach to reaction mechanism that brings together metathesis reactions involving the formation of a variety of metal‐element bonds through partner interchange of σ‐bond complexes. It is supported through experimental measurements and computational studies of stoichiometric and catalytic reactions that are becoming increasingly sophisticated.[^147^, ^150^, ^151^] The key concept that defines a metathesis reaction as a σ‐CAM process is the presence of two σ‐bond complexes as intermediates. They must retain the metal in the same oxidation state and must be connected by a *single* transition state. The nature of this transition state, however, does not define whether it is a σ‐CAM process or not. This definition allows for experimental and computational investigation of the intermediates, while allowing flexibility and nuance in the nature of the transition states.

## Conflict of interest

The authors declare no conflict of interest.

## Biographical Information

*Robin Perutz is an emeritus professor at the University of York. During his PhD under J. J. Turner in Cambridge and Newcastle, Robin revealed the formation of metal methane complexes. Subsequently, he worked in Mülheim, Edinburgh and Oxford, before moving to York in 1983. Robin aims to understand the activation of small molecules and the mechanisms of catalytic reactions, often through photochemical reactions. He has received awards from the Royal Society of Chemistry, the Italian and the French Chemical Societies. He was elected a Fellow of the Royal Society, the UK′s national academy of sciences, in 2010. Robin has been active in the women in science agenda. He is also concerned with protecting human rights of scientists fleeing conflict*.

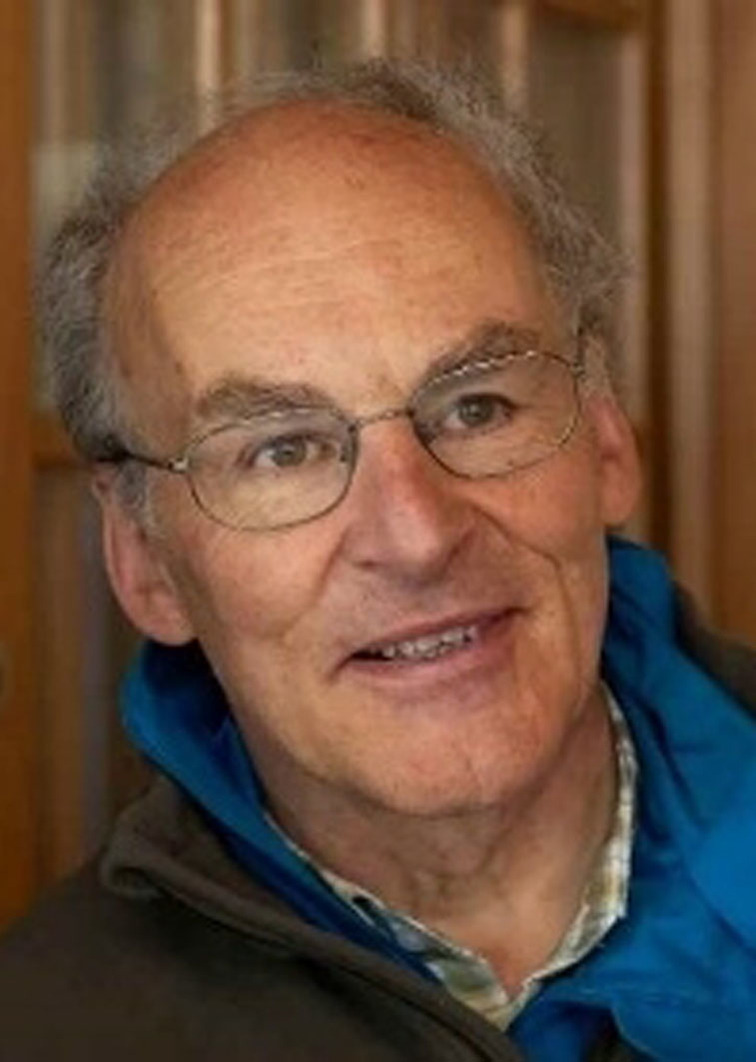

## Biographical Information

*Sylviane Sabo‐Etienne has just retired as CNRS Director of Research Exceptional Class. She spent most of her career at the Laboratoire de Chimie de Coordination in Toulouse, France. Her broad research interests encompass coordination chemistry and organometallic chemistry for applications in the fields of energy and catalysis with a specific focus on polyhydrides and dihydrogen transition metal complexes. She is now active in promoting projects combining arts and sciences with a strong commitment to audiences unfamiliar with these cultures and to those with disabilities*.

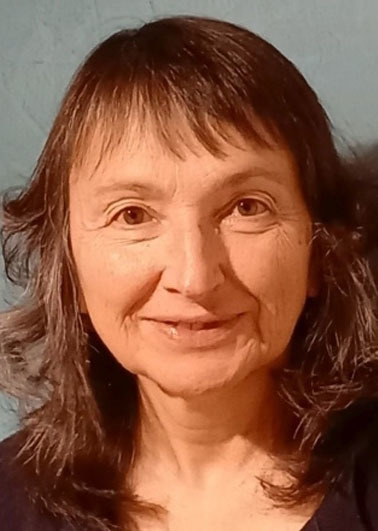

## Biographical Information

*Andrew Weller is Professor of Inorganic Chemistry at the University of York. Prior to this he was Professor of Chemistry at the University of Oxford, after starting his independent career at the University Bath as a Royal Society University Research Fellow. His first degree was from University of Warwick, a PhD from Bristol (John Jeffery), and PDRA positions at Heriot‐Watt (Alan Welch) and Notre Dame (Tom Fehlner). Research in the Weller group is based upon synthetic organometallic chemistry and catalysis, and in particular transition metal complexes that have structures that display C−H, B−H and C−C bonding modes with metal centers*.